# Synthesis of an Ethylenediaminetetraacetic Acid-like Ligand Based on Sucrose Scaffold and Complexation and Proton Relaxivity Studies of Its Gadolinium(III) Complex in Solution

**DOI:** 10.3390/molecules29194688

**Published:** 2024-10-03

**Authors:** Ping Zhang, Cécile Barbot, Ramakrishna Gandikota, Cenxiao Li, Laura Gouriou, Géraldine Gouhier, Chang-Chun Ling

**Affiliations:** 1 Department of Chemistry, University of Calgary, 2500 University Drive NW, Calgary, AB T2N 1N4, Canada; zhap@ucalgary.ca (P.Z.); cenxiao.li@ucalgary.ca (C.L.); 2 University Rouen Normandie, INSA Rouen Normandie, CNRS, Normandie University, COBRA UMR 6014, INC3M FR 3038, F-76000 Rouen, France; cecile.barbot@univ-rouen.fr (C.B.); ramakrishna-kishore-kumar.gandikota@univ-rouen.fr (R.G.); laura.gouriou2@univ-rouen.fr (L.G.)

**Keywords:** sucrose, aminopolycarboxylate, lanthanide complex, stability constant, proton relaxivity

## Abstract

Sucrose constitutes a non-toxic, biodegradable, low-cost and readily available natural product. To expand its utility, we developed total synthesis for a ligand based on a sucrose scaffold for potential use as a metal chelation agent. The designed target (compound **2**) has a metal-chelating functionality at both the C-6 and C-6’ positions, which can provide a first coordination sphere of eight valencies. The designed total synthesis was highly efficient. To demonstrate the utility of the ligand, we studied its complexation with Gd(III). Using potentiometric titration and high-resolution mass spectrometry, we confirmed the formation of a 1:1 complex with Gd(III), which has a respectable formation constant of ~10^13.4^. Further NMR relaxivity studies show that the Gd(III) complex has a relaxivity (r1) of 7.6958 mmol^−1^ s^−1^.

## 1. Introduction

**Figure 1:**
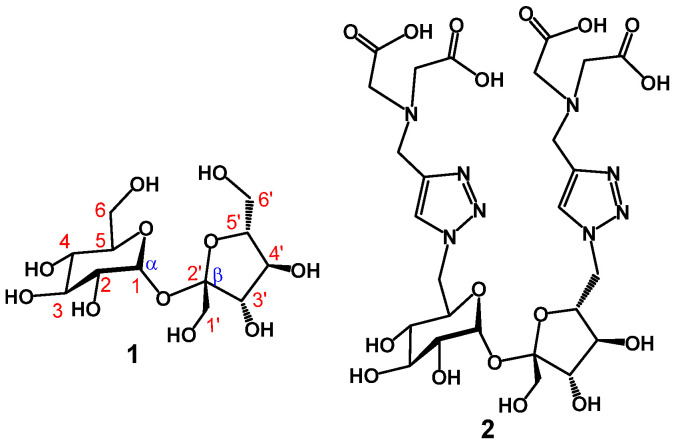
Structures of sucrose disaccharide (**1**) and newly designed synthetic ligand (**2**) using sucrose as a scaffold.

Sucrose or table sugar (**1**, Figure 1) is an inexpensive agricultural product primarily produced by sugarcane and sugar beet [1]. With an annual production of more than 180 million metric tons, sucrose constitutes a genuinely green, biorenewable and sustainable natural product. Due to its abundance and extremely low cost, sucrose has found many utilities in different fields, in addition to being primarily consumed as a natural sweetener to foods and other agricultural products, medicines and cosmetics [2]. For example, sucrose has been converted into biodegradable polymers [3,4,5], such as polyesters and polyurethanes, and other value-added commercial products, such as emulsifiers [3,6,7,8], fat substituents [9] and artificial sweeteners [10,11]. With the help of fermentation and technologies developed in chemical industries, sucrose has also found other important utilities, such as bio feedstock for the production of biofuels and others [12,13]. Structurally, sucrose is a disaccharide that is formed between α-D-glucopyranose and β-D-fructofuranose by condensing their anomeric hydroxyls together to form an α(1→2’)glycosidic linkage (**1**, Figure 1). This makes sucrose a non-reducing sugar, which represents an advantage in chemical functionalization as it exists as a single stereoisomer. There are three primary hydroxyl groups at C-6, C-1’ and C-6’ that can be selectively activated, with two of the three hydroxyl groups (OH-6 and OH-6’) being even more accessible than the third one because of their reduced steric hindrance. As a result of these chemical features, sucrose has become a valuable scaffold for designing crown ethers, cryptands and other macrocycles [3]. In this work, we wish to report another possible application that uses sucrose as a scaffold to design metal-binding aminopolycarboxylates [14], such as compound **2** (Figure 1), for chelating transition metal ions. Compound **2** is strategically functionalized with two iminodiacetate residues, respectively, at the C-6 and C-6’ positions with the help of copper(I)-catalyzed alkyne-azide 1,3-dipolar cycloaddition (CuAAC) [15,16]. The two iminodiacetate residues with the two newly formed 1,2,3-triazole rings can potentially work together by providing eight coordinating sites as the first coordination sphere for lanthanides, such as gadolinium(III), whose complexes have been widely used in magnetic resonance imaging [17,18]. The hard acid Gd^3+^ favors basic donor atoms, such as nitrogen and charged oxygen, which explains why many ligands containing nitrogen and oxygen are used as chelating and decontaminating agents, such as EDTA (ethylenediaminetetraacetic acid), NTA (nitrilotriacetic acid) and EGTA (ethylene glycol-bis(2-aminoethyl ether)-*N*,*N*,*N’*,*N’*-tetraacetic acid). 

## 2. Results and Discussion

### 2.1. Chemical Synthesis and Characterization

**Scheme 1:**
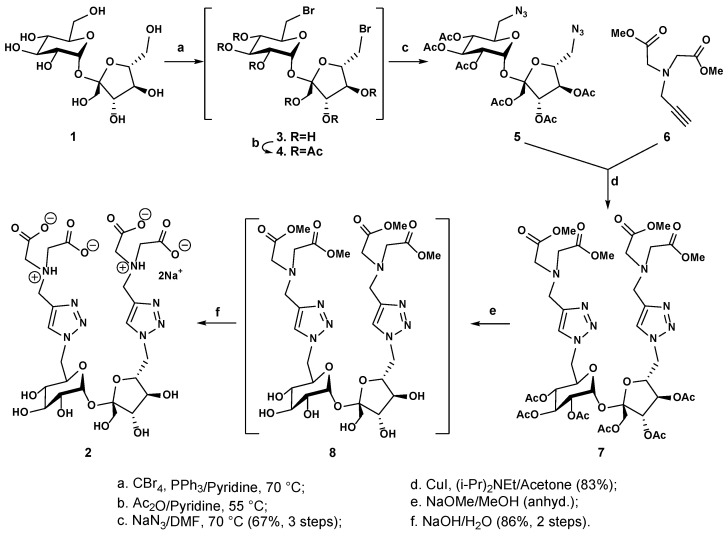
Synthetic route to the designed target ligand (**2**) from sucrose (**1**).

The targeted ligand (**2**) was synthesized by following a highly efficient five-step route, outlined in Scheme 1, using the commercially available sucrose (**1**) as a starting material. The hydroxyl groups at C-6 and C-6’ positions were regioselectively activated with dibromination, using carbon tetrabromide and triphenylphosphine as the reagents at 70 °C in anhydrous pyridine. This did not affect the other more sterically hindered primary hydroxyl group at C-1’. The formed C-6,C-6’-dibromide (**3**) was not isolated but directly subjected to per-*O*-acetylation at 55 °C to afford crude per-*O*-acetylated 6,6’-dibromide (**4**), which again was not isolated but subjected to substitution with sodium azide in *N,N*-dimethylformamide at 70 °C overnight. This afforded the corresponding 6,6’-diazide (**5**), which was isolated in its pure form and had a very good yield (67% over three steps). The recorded ^1^H NMR spectrum of compound **5** showed two sets of mutually coupled protons at a fairly shielded region, each of them appearing as a doublet of doublets (Figure S1). They were assigned to be the two sets of geminal protons, respectively, attached to azido-functionalized C-6’ of the fructofuranosyl unit [3.62 ppm (*J* = 13.1, 4.2 Hz)/3.48 ppm (*J* = 13.1, 4.2 Hz)] and azido-functionalized C-6 of the glucopyranosyl unit [3.36 ppm (*J* = 13.1, 7.5 Hz)/3.27 ppm (*J* = 13.4, 5.9 Hz)], according to the 2D ^1^H-^1^H GCOSY correlation spectrum (Figure S3). On the other hand, an AB quartet was observed to be integrated for two protons at a more deshielded region, corresponding to the set of geminal protons attached to C-1’ of the fructofuranosyl unit [4.19 ppm (*J* = 12.3 Hz) and 4.13 ppm (*J* = 12.3 Hz)]. The fact that these two protons were observed to be more deshielded (>4 ppm) is consistent with the attachment of a more electron-withdrawing *O*-acetyl group. Furthermore, the recorded ^13^C NMR spectrum (DEPTQ, Figure S2) and 2D ^1^H-^13^C GHSQC heteronuclear correlation spectrum (Figure S4) revealed one primary carbon signal at 62.5 ppm (C-1’), which was more deshielded than the other two primary carbons at 52.7 (C-6’) and 51.0 (C-6) respectively, further confirming that C-1’ has an *O*-acetyl group attached, while both C-6’ and C-6 are attached to an azide functional group. 

With the fully protected 6,6’-diazide (**5**) in hand, we proceeded with the coupling reaction with the previously reported dimethyl *N*-propargyl iminodiacetate (**6**) [18] using copper(I) iodide as a catalyst and *N,N*-diisopropylethylamine as a base; this successfully yielded the corresponding conjugate **7,** which was isolated in its pure form and had a very good yield (83%). The structural identity of compound **7** was confirmed by the presence of two singlets at 7.67 and 7.57 ppm, respectively (Figure S5); these two aromatic protons correspond to H-4 protons of the two newly formed 1,2,3-triazole rings. The two sets of geminal protons, respectively, attached to C-6’ and C-6 shifted to a more deshielded region (4.45/4.30 ppm for H-6a’/H-6b’ and 4.45/4.37 ppm for H-6a/H-6b) as a result of their proximity to the 1,2,3-triazole rings that have a deshielding effect due to their aromatic π-electron clouds. 

**Figure 2:**
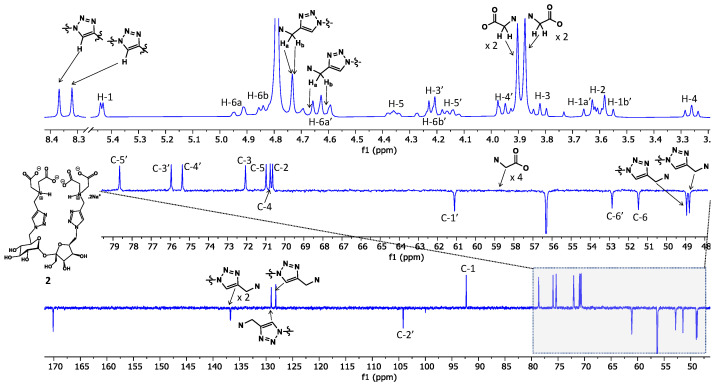
Recorded NMR spectra and signal assignments of compound **2** in D_2_O. Top: 1D ^1^H spectrum (400 MHz); bottom: 1D ^13^C spectrum with the expansion of the ~48.0–79.5 ppm region.

**Figure 3:**
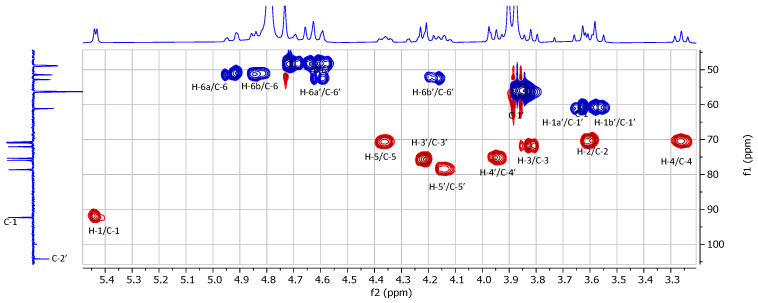
Recorded 2D ^1^H-^13^C heteronuclear correlation NMR spectrum (400 MHz) of compound **2** in D_2_O.

To obtain the final target (**2**), compound **7** was first subjected to per-*O*-deacetylation in anhydrous methanol using a catalytic amount of sodium methoxide as a base. Trans-esterification afforded the intermediate (**8**), which was not isolated but directly subjected to full saponification with aqueous sodium hydroxide to afford the desired compound (**2**), isolated in sodium salt form by size-exclusion chromatography on Sephadex LH-20 using methanol as the eluent (86% yield). The structure of compound **2** was confirmed by 1D ^1^H and ^13^C spectra (Figure 2, Figures S9 and S10), and the observed ^1^H and ^13^C signals were assigned with the support of 2D ^1^H-^1^H homonuclear GCOSY (see Figure S11) and ^1^H-^13^C heteronuclear GHSQC (Figure 3 and Figure S12) correlation spectra. As can be seen in the H-1 spectrum recorded in D_2_O, the anomeric proton of the D-glucopyranosyl unit is observed at 5.44 ppm as a doublet with a small coupling constant (*J* = 3.7 Hz), corresponding to its α-anomeric configuration; this proton correlates to a carbon at 92.3 ppm (C-1), according to the GHSQC heteronuclear correlation spectrum. On the other hand, for the fructofuranosyl unit, the anomeric center C-2’ can be observed at 104.2 ppm; there is no anomeric proton attached to C-2’, as evidenced by the absence of correlation with C-2’ from any protons in the GHSQC spectrum. The two geminal protons at C-1’ of the fructofuranose are observed at 3.64 and 3.57 ppm as two mutually coupled doublets with a large coupling constant (*J* = 12.6 Hz). There are two sets of 1,2,3-triazole units, as evidenced by the two singlets at 8.37 and 8.32 ppm, corresponding to the H-4 aromatic protons. The two pairs of iminoacetates attached to C-6 (Glu) and C-6’ (Fru) are observed as two separate singlets at 3.90 and 3.88 ppm (two methylene protons) in the 1D proton NMR spectrum, suggesting that they have different chemical environments due to free rotation around the parent C-N single bond from the 1,2,3-triazolmethyl residue. On the other hand, we noticed that all methylene protons of the two iminoacetates shifted to a significantly more deshielded region compared to those of compound **7** (3.48 and 3.44 ppm), suggesting that both the tertiary amine centers of the iminoacetates in isolated compound **2** are likely to be protonated. The structure of compound **2** was further confirmed by electrospray high-resolution mass spectrometry (HRMS, negative), which showed a peak at *m*/*z* 733.2289 and another peak at *m*/*z* 366.1114, corresponding to the expected mono- and double-charged molecule ions that, respectively, have a molecular formula of C_26_H_37_N_8_O_17_ [M-H]^−^ (calculated *m*/*z*: 733.2282) and C_26_H_36_N_8_O_17_ [M-2H]^2−^ (calculated *m*/*z*: 366.1105). 

### 2.2. Protonation Studies of Compound **2** by Potentiometric Titration 

As alluded to above, the structure of ligand **2** contains four carboxylate residues and two tertiary amine centers. Depending on the pH of the media, it can exist in different protonation states. Based on the well-known pKa values for acetic acid (~4.76) and triethylammonium (~10.75) and a literature report on the pKa of the protonated 1,2,3-triazolium compound, *N*-methyl-1,2,3-triazolium (~1.25) [19], we can safely ignore the protonation of the two 1,2,3-triazole rings in compound **2** since it only becomes relevant at very acidic pHs. Thus, in less acidic solutions, ligand **2** contains essentially six prominent protonation sites that include the two tertiary amine centers and four carboxylates. 

**Scheme 2:**
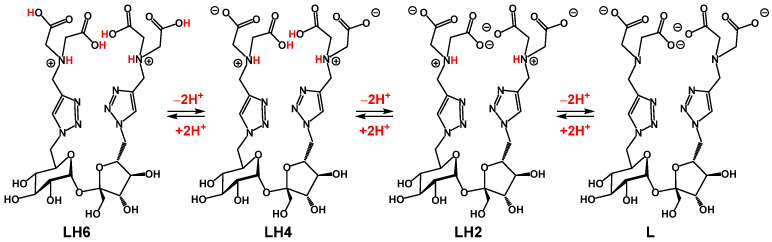
The major protonation states of target ligand **2** and their designated notations.

To facilitate the discussion of our potentiometric titration results, the following notations were adopted (Scheme 2): L represents the form of a compound with full deprotonation of all the above sites, LH2 represents the species with essentially full deprotonation of all four carboxylate groups, while the two tertiary amine centers remain protonated, and LH6 represents the form of compound **2** with protonation of all the four acetates and two tertiary amine centers. Other notations will be used accordingly to designate intermediate deprotonation states. 

**Figure 4:**
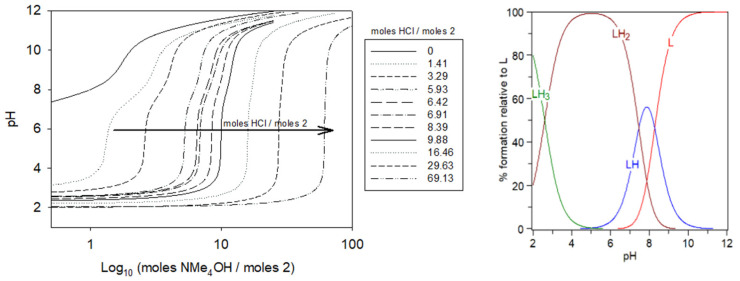
Potentiometric titrations of compound **2** (**left**). L: 0.5–3 µmol; extra HCl: 0–34.5 µmol in 0.1 M NMe_4_Cl; total initial volume: 4.0 mL. Burette: [NMe_4_OH] = 0.05 M, and calculated distribution curves (**right**) of different protonated species of compound **2** at different pHs: [compound **2**] = 5.0 × 10^−4^ M.

As the pH of the initial solution of isolated compound **2** in water was more than 7, we concluded that at this elevated pH, the four carboxylates are most likely deprotonated; this supports that the isolated compound structure is in the LH2 form, as suggested by the ^1^H NMR experiment. We gradually added a solution of HCl in trimethylammonium chloride (0.1 M) to the initial solution to obtain a series of solutions with different HCl/compound **2** ratios (0→69.1) while maintaining the total volume constant (4.0 mL). To each prepared solution, potentiometric titrations were initiated using a titrant solution of tetramethylammonium hydroxide (0.05 M); the pHs of the solutions were recorded. This allowed us to obtain a series of titration curves (Figure 4). As can be seen, with increasing HCl/**2** ratios, the form of the obtained curves becomes increasingly similar to that of pure HCl, while with decreasing HCl/**2** ratios, the form of the curves becomes more representative of ligand **2** alone.

The protonation constant *β_h_* for the following equilibrium is defined by Equation (1), where L represents the fully deprotonated form, and H is the proton (charges are omitted).

(1) $$L+\mathrm{hH}\;⇌\;{\mathrm{LH}}_{h};\;\beta_{h}=\frac{\left[{LH}_{h}\right]}{{\left[H\right]}^{h}[L]}$$

As dissociation constants, *Ka* values are commonly defined by:

(2) $$K_{a}=\frac{\left[H][{LH}_{h-1}\right]}{\left[LH_{h}\right]}$$

It is evident that:

(3) $${pK}_{a}=\log\beta_{h}-log\beta_{h-1}$$

Table 1 summarizes all the protonation constant values of compound **2** determined by titration curve refinement, together with the corresponding acidity constants. All the *pKa* values were ascribed to deprotonation of the carboxylate functions, followed by ammonium functions when increasing the base addition.

The last carboxylate group to be deprotonated, named LH3, had a *pKa* (LH3/LH2) equal to 2.6. The *pKa* values greater than 7 could be attributed to the formation of ammonium moieties, which is in agreement with the known values for these groups (LH2/LH: 7.46; LH/L: 8.28).

**Scheme 3:**
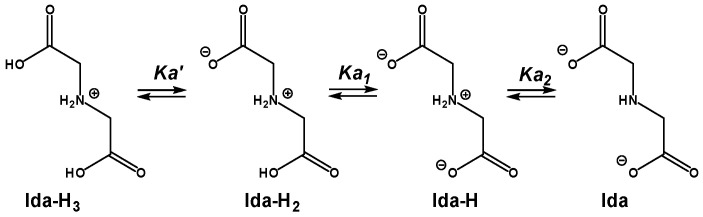
The three protonation states of iminodiacetic acids.

The obtained *pKa* values are in accordance with the literature for iminodiacetic acid (Ida) in an aqueous medium (Scheme 3) at the same ionic strength (0.1 M): *pKa_1_* = 2.68 in the case of the equilibrium Ida-H_2_/Ida-H [20]. For the Ida-H_3_/Ida-H_2_ equilibrium, the reported *pKa* was 1.84, which is very low, and we were not able to reach it in our experiments. 

However, the reported *pKa_2_* accounting for ida-H/ida was 9.47, which is less acidic than the LH/L value of 8.28. This can be explained by the difference in amino functionality, which, in our case, is linked to a triazole moiety through an intermediate methylene group. As the triazole ring is a strong electron-attracting group, this can slightly lower the *pKa* of LH species compared to that of iminodiacetic acid. 

From the species diagram of compound **2** (Figure 4, right), at physiological pH, the majority of species would be LH2 in equilibrium with LH. Therefore, we conclude that all the carboxylate groups were deprotonated. This is consistent with our hypothesis based on the NMR experiment.

### 2.3. Complexation Studies of Gadolinium(III) with Compound **2** by High-Resolution Mass Spectrometry 

**Figure 5:**
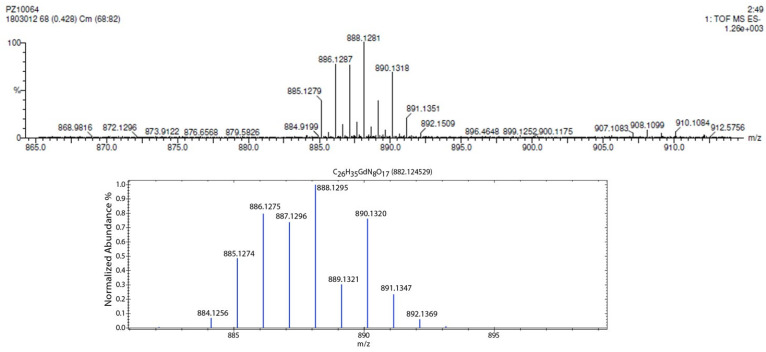
Electrospray ionization (negative) high-resolution spectrum (**top**) of formed 1:1 complex [GdL]^−^ with a molecular formula of C_26_H_34_N_8_O_17_Gd [M-H]^−^ between ligand **2** and Gd(III), and comparison to the isotope patterns of simulated mass spectrum (**bottom**).

To determine the formation of the complex between compound **2** and lanthanide metals, we selected gadolinium(III) as a model. A solution of compound **2** was mixed with a gadolinium(III) trichloride solution (1.1 equivalents). The pH of the solution was then adjusted to 6~7, and the product formed was dialyzed against deionized water several times using a membrane with a molecular weight cut-off of ~500 daltons; this permits the removal of impurities with a molecular weight < 500 daltons. The solution was lyophilized to afford the ligand **2**/Gd(III) complex as a white solid. Unfortunately, due to the polyhydroxylated nature of the ligand, we were unable to find conditions to grow single crystals for structural determination by X-ray diffractometry. Additionally, due to the paramagnetic nature of Gd^3+^ ions, we were also unable to characterize the formed complex by NMR spectroscopy. However, the obtained solid was successfully analyzed by high-resolution mass spectrometry (electrospray, negative, Figure 5, top). The mass spectrum revealed a cluster of peaks centered at *m*/*z* 888.1281 (100%), together with other peaks. These peaks and the associated isotope patterns matched very well with the simulated spectrum (Figure 5, bottom) of the expected 1:1 complex [GdL]^−^ between compound **2** and Gd(III) ions and has a molecular formula of C_26_H_34_N_8_O_17_Gd [M-H]^−^ (calculated *m*/*z*: 888.1295 (100%)). This evidence confirms the successful formation of the 1:1 complex [GdL]^−^. 

### 2.4. Studies of Ligand **2**/Gd(III) Complex by Attenuated Total Reflectance–Fourier Transform Infrared (ATR-FTIR) Spectroscopy 

**Figure 6:**
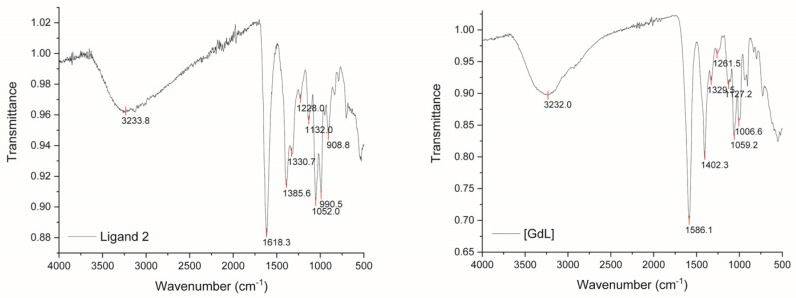
ATR-FTIR spectra of ligand **2** (**left**) and ligand **2**/Gd(III) complex (**right**).

In order to gain insight into the involvement of carboxylates in coordination with the Gd(III) metallic center, Attenuated Total Reflection–Fourier Transform Infrared (ATR-FTIR) spectroscopy was used to study ligand **2** and its complex with Gd(III). It is well-known that deprotonation of carboxylic acid results in the absence of strong bands around 1700 cm^−1^ [21,22]. The resulting carboxylate (COO^−^) usually has two vibrational modes around 1600 and 1400 cm^−1^ due to symmetric and antisymmetric stretching. As can be seen from Figure 6 (left), the ATR-FTIR spectrum of ligand **2** showed two stretching bands at 1618.3 and 1385.6 cm^−1^, indicating the isolated ligand was, indeed, fully deprotonated from the carboxylates. Upon complexing with Gd(III), the peak corresponding to the asymmetric mode (1618.3 cm^−1^) shifted to lower wavenumbers (1586.1 cm^−1^), indicating that coordination through the carboxylate groups takes place (Figure 6).

### 2.5. Complexation Studies of Gadolinium(III) with Ligand **2** by Potentiometric Titration 

To gain a deeper understanding of the complex formed between ligand **2** and gadolinium(III) in an aqueous solution, we next determined the stability constant using potentiometric titration. A series of solutions containing 0 to 1.11 equivalents of Gd(III)/ligand **2** were prepared by maintaining the initial concentrations of ligand **2** (5 × 10^−4^ M) and HCl (3.45 × 10^−3^ M) constant. Similarly, to each prepared solution, potentiometric titrations were performed by adding a titrant solution of tetramethylammonium hydroxide (0.05 M). The pH of the solution was recorded. 

With M being the metal ion, L being the ligand, and H being the proton, the stability constants of the complexes with the general formula $M_{m}L_{l}H_{h}$ are expressed by the following equations (the charges are omitted):

(4) $$mM+lL+hH⇌M_{m}L_{l}H_{h};\;\beta_{h}=\frac{\left[M_{m}L_{l}H_{h}\right]}{{\left[M\right]}^{m}{\left[L\right]}^{l}{\left[H\right]}^{h}}$$

Thus, the dissociation constant *K_mlh_* can be defined similarly to the case of the free ligand, as follows:

(5) $$K_{mlh}=\frac{\left[H][M_{m}L_{l}H_{h-1}\right]}{\left[M_{m}L_{l}H_{h}\right]}$$

Analogously,

(6) $$pK_{mlh}=\log\beta_{mlh}-log{\;\beta }_{mlh-1}$$

**Figure 7:**
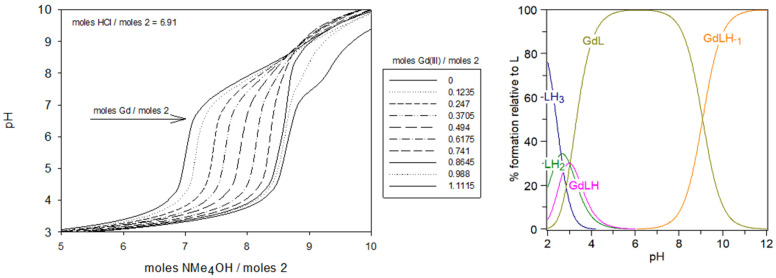
Potentiometric titrations of ligand **2** in the presence of gadolinium(III) (**left**). Seventeen titrations were performed. Ligand **2**: 2.0 µmol; extra HCl: 13.82 µmol in NMe_4_Cl (0.1 M); total initial volume: 4.0 mL. Burette: [NMe_4_OH] = 0.05 M. The calculated distribution curved of 1:1 complex during titration [Gd]t = [Ligand **2**] = 5.0 × 10^−4^ M (**right**).

Figure 7 shows the obtained titration curves of the complex formed between ligand **2** and Gd(III). As can be seen, the GdL complex was formed over pH 3 as soon as the carboxylate moieties were deprotonated. For each solution, the pH slowly increased with the addition of the base; however, the pH of the solution with the greatest Gd:L ratio increased the slowest when the same amounts of the base were added. This can be explained by the fact that during the complexation of gadolinium(III), protons were released from the protonated sites of ligand **2**, acidifying the medium. As shown in Figure 4, at pH 5, the speciation of the ligand alone has LH2 as the predominant species that corresponds to the form containing two protonated tertiary amines and four fully deprotonated carboxylate groups. At the same pH, in the presence of Gd(III), the titration necessitated about 2 moles of the base/mol of GdCl_3_ to be added, and the speciation diagram indicates that the stoichiometry of the Gd:ligand **2** complex to be 1:1 as the predominant species (GdL, Figure 7), supporting the removal of the two protons from the tertiary amines during complexation. Under physiological conditions (pH 7.4), there is an equilibrium between LH2 and LH of about equal proportions (Figure 4). This pH corresponds to the second pKa of ligand **2** (Table 1). As shown in Figure 7, upon complexation at pH 7.4, the predominant species of Gd:ligand **2** remains 1:1 (i.e., GdL). In this case, complexation requires about 1.5 moles of the base/mol of gadolinium(III), corresponding to ~50% deprotonation of LH2. Finally, when the pH was well over the third pKa = 8.28, the fully deprotonated form (L) became the predominate species in equilibrium with LH (Figure 4), thus the volumes of the base added were similar for the gadolinium complex and ligand alone, as there were no more protons to be removed upon complexation.

**Scheme 4:**
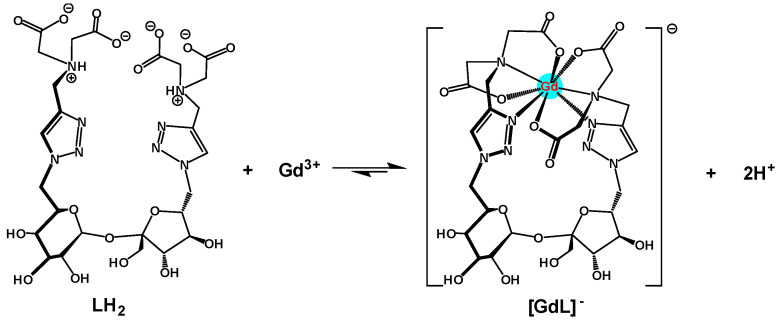
Suggested complexation reaction of ligand **2** with Gd(III) ion results in the release of protons.

Consequently, considering the whole results obtained for the ligand **2**/Gd(III) complex, the best chemical model that fits with the potentiometric curves for complexation corresponds to the formation of the essentially mononuclear GdL species with a ratio of 1:1 (Scheme 4); this represents more than 50% of the total species above pH 4, as illustrated in the calculated distribution curves (Figure 7, right). At pH 7.4, under physiological conditions, 98% of the ligand is on the GdL form, and 2% is on the GdLH-1 form. We can assess that, under these conditions, the complexation of Gd(III) was effective. When the Gd(III) concentration was higher than that of ligand **2**, precipitation occurred; this can be explained by the formation of hydroxylated species, such as Gd(OH)_3_.

The stability constants of the ligand **2**/Gd(III) complexes are reported in Table 2. The formation constant for the monometallic complex logβ_GdLH_ was determined to be 13.4. This value is weaker than those reported for other aminocarboxylate ligands, such as EDTA (log βGd = 17.7) [23], and FDA-approved contrast agents (CAs), such as DTPA Magnevist^TM^ (log βGd = 22.1), DOTA Dotarem^TM^ (log βGd = 25.6) and DTPA.bma Omniscan^TM^ (log βGd = 16.9) [24]. It is also lower than that of the cyclodextrin ligand, which we reported previously (log βGd = 25.09) [18]. However, the stability constant of the ligand **2**/Gd complex is much higher than those described for the native cyclodextrins/Gd(III) (log βGd = 2.5) [25] or tris(hydroxymethyl)aminomethane (Tris)/Gd(III) (log βGd = 2.67) complexes [26]. In comparison with Tris, the presence of *N*-acetate residues in ligand **2** clearly shows advantages as a result of the introduction of two units of iminodiacetate groups; using sucrose as a scaffold to support the two fragments of iminodiacetates further allows an effective collaboration during the complexation of Gd(III). However, the larger macrocycle in the formed complex of ligand **2** also introduces enhanced conformation flexibility, thus accounting for the reduced stability compared to other ligands. On the other hand, the glycosidic linkage of ligand **2** represents an advantage because it is acid-labile and enzyme-active; thus, ligand **2** can be readily degraded and used as an environmentally friendly chelating reagent. 

**Figure 8:**
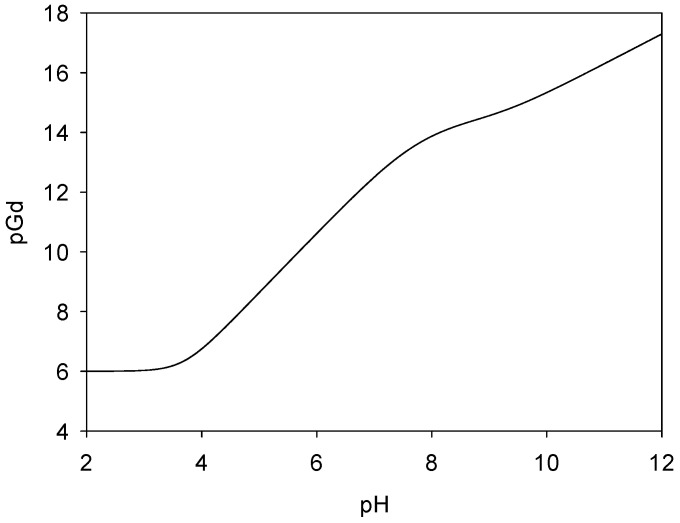
p*Gd* value of Gd(III)-ligand **2** complexes as a function of pH. p*Gd* = −log[Gd]_free_, [Gd]_total_ = 1.0 µM, and [ligand]_total_ = 10 µM.

The p*M* value measures the concentration of uncomplexed free metal ions left in the solution by a ligand. The variation in p*M* with pH for a particular metal–ligand complex can give useful information about the stability of the complex under different pHs; thus, the p*M* value provides a more complete picture of the effectiveness of a ligand when chelating a metal ion. The p*Gd* (where p*Gd* = −log[Gd_free_]) was calculated as a function of pH for a standard set of conditions (initial concentration: [Gd] = 10^−6^ M, [L] = 10^−5^ M) and is represented in Figure 8.

The p*Gd* needs to be as high as possible to ensure negligible dissociation, as Gd(III) is toxic. The higher the p*Gd* of a complex, the better affinity the ligand has toward the metal center. In the case of the current complex studied, the maximum p*Gd* value (>14.5, Figure 8) is obtained at pH 9. Under physiological conditions (pH 7.4), p*Gd* was determined to be ~13.1. When compared to the p*Gd* values reported in the literature for other Gd(III) complexes, such as Gd-DOTA (p*Gd*~18.1) [27], a tightly caged complex, and our previously reported 1:1 cyclodextrin ligand/Gd(III) complex (p*Gd*~15.60) [18], the p*Gd* value for the ligand **2**/Gd(III) complex is lower but still quite impressive, considering its larger and more flexible macrocycle and noncaged architecture. 

### 2.6. Relaxivity Measurement of Ligand **2**/Gd(III) Complex 

**Scheme 5:**
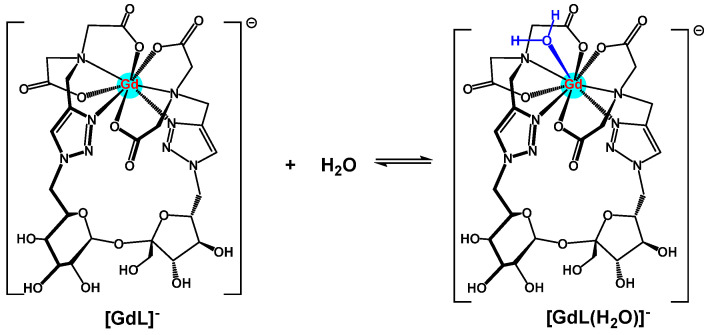
Suggested dynamic formation of aquacomplex of ligand **2**/Gd(III) complex with water (pH < ~7.5).

Lanthanides are well-known to be capable of nine coordinations. The current ligand (**2)** can provide eight coordinating sites from two iminoacetates plus two 1,2,3-triazole units; this leaves the gadolinium(III) center to offer one additional site for dynamic water coordination to form the aquacomplex [GdL(H_2_O)]^−^ (Scheme 5) [18]. However, over pH ~7.5, hydroxocomplexes appeared for different ratios of Gd:ligand. In this case, the hydroxide ligand likemly replaces the aqua ligand in the coordination sphere to form [GdL(OH)]^−2^, as observed in the titration curves, with an inflection point occurring between pH 7 and 8.

**Figure 9:**
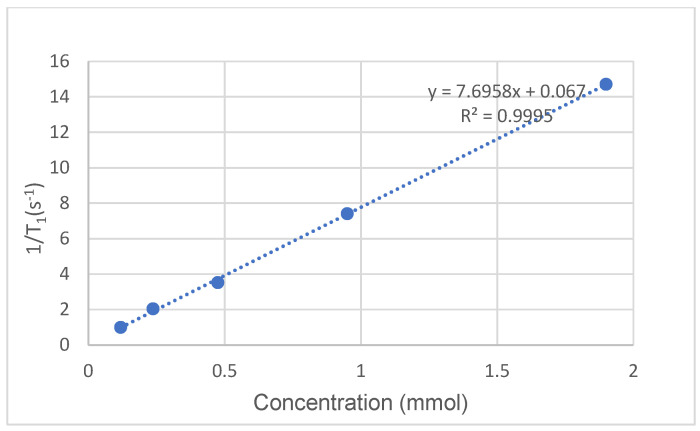
The plot of 1/T_1_ versus the concentration of ligand **2**/Gd(III) complex gives a linear line with a slope of 7.6958 mM^−1^s^−1^.

Relaxivity (T_1_) measurements were performed on the ligand **2**/Gd(III) complex at 37 °C on a 0.47 T (20 MHz) instrument using the “Inversion Recovery Pulse Sequence”. The results are summarized in Table 3 and plotted in Figure 9. The obtained curves were adjusted to a monoexponential function to obtain T_1_. Relaxation values were measured three times, and the average was calculated.

The slope of the fitted line of 1/T_1_ versus the concentration of the complex leads to the relaxivity value (r_1_), which has a unit of mM^−1^s^−1^. Based on the results, the calculated relaxivity of ligand **2**/Gd(III) is 7.6958 mM^−1^s^−1^ (R^2^ = 0.9995), which is actually much better than the reported relaxivity of the DOTA/Gd(III) complex (r_1_ = 3.2 mM^−1^s^−1^) [28,29].

The size and molecular weight of the paramagnetic complex can affect the rotational correlation time (τ_r_) and the residence time of water molecules (τ_m_) in the sphere of coordination of gadolinium(III) [17]. Larger molecules have a slower rotation, thus increased τ_r_ and faster exchange rate k_ex_ decreases of τ_m_, thus improving the relaxivity. The difference in relaxivity of ligand **2**/Gd(III) compared to the DOTA/Gd(III) complex can be explained by their large differences in molecular weights (*m*/*z*: 888.13 for ligand **2**/Gd(III) versus *m*/*z*: 558.65 for DOTA-Gd(III)) as well as the presence of several hydroxyl groups in ligand **2**, which can potentially interact with water molecules via hydrogen bonding, forming a second coordination sphere. In the DOTA/Gd(III) complex, there is a lack of hydroxyl groups; thus, similar hydrogen bonding with water molecules is absent. In a similar way, higher relaxivity has been observed with the native β-cyclodextrin/Gd(III) complex in comparison with the permethylated β-cyclodextrin/Gd(III) complex, confirming the importance of hydrogen bonding interaction in T_1_ measurement [17]. 

## 3. Conclusions

Sucrose is a highly abundant, low-cost, non-toxic, eco-friendly natural product. We reported a potential application by converting sucrose to useful metal-chelating ligands. The chemistries reported in this work are highly regioselective for both C-6 and C-6’ positions, and we demonstrate that two iminoacetate residues can be easily installed on those two positions by taking advantage of the efficient “click” reaction. We further demonstrate that the introduced functionalities can effectively collaborate to form a multidentate first-coordination sphere for lanthanide metal ions. Using gadolinium(III) as a model, we provide evidence that the formed complexes (such as ligand **2**) can achieve respectable binding affinity and higher relaxivity. However, because of its lower p*Gd* compared to the Gd-DOTA complex currently used in clinics, it is unlikely for ligand **2** to find clinical applications as a contrast agent in MRI. However, its ability to sequester metal ions can be extended to many other metals, and we, thus, expect ligands based on sucrose scaffolds, such as compound **2,** to find utility in many other applications.

## 4. Materials and Methods

### 4.1. Chemical Synthesis 

All commercial reagents were used as supplied unless otherwise stated. Analytical thin layer chromatography was performed on Silica Gel 60-F_254_ TLC Plates (Sigma-Aldrich^®^, Oakville, ON, Canada) with detection by charring with 5% sulfuric acid in water or a ceric ammonium molybdate dip. Column chromatography was performed on Silica Gel 60 (Silicycle, Ontario, Canada). Organic solutions from extractions were concentrated under vacuum with the assistance of a heat bath. Fourier Transform–InfraRed Spectroscopy was obtained on a Bruker Alpha routine FT-IR spectrometer with ALPHA’s Platinum ATR single reflection diamond module in the analysis range and wavenumber from 387 up to 4000 cm^−1^ in the transmittance mode with 1 s per scan and data collection of 24 scans per analysis. ^1^H NMR spectra were recorded at 400 MHz, and ^13^C NMR spectra were recorded at 101 MHz on a Bruker spectrometer. Chemical shifts *δ*_H_ and δ_C_ were reported in *δ* (ppm) and referenced to the residual CHCl_3_ (*δ*_H_ 7.24, *δ*_C_ 77.0, CDCl_3_) and residual HDO (*δ*_H_ 4.79) of the D_2_O solvent and external acetone (*δ*_C_ 29.9). First-order coupling constants were reported in Hz for proton nuclei. ^1^H and ^13^C NMR spectra were assigned with the assistance of DEPTQ, GCOSY and GHSQC spectra. High-resolution ESI-QTOF mass spectra were recorded on an Agilent 6520 Accurate Mass Quadrupole Time-of-Flight LC/MS spectrometer.

**2,3,4,1**′**,2**′**,3**′**-Hexa-*O*-acetyl-6,6**′**-diazido-6,6**′**-dideoxysucrose (5).** Sucrose (**1**, 2.02 g, 5.90 mmol) was added to anhydrous pyridine (30.0 mL), and the mixture was heated to 110 °C to allow all solids to dissolve. The solution was cooled to an ambient temperature, and Ph_3_P (4.61 g, 17.5 mmol) was added. This was followed by the dropwise addition of a solution of CBr_4_ (5.82 g, 17.5 mmol) in anhydrous pyridine (10.0 mL). The mixture was then heated to 70 °C for 2 h to form the intermediate 6,6’-dibromide (**3)** as the major product (based on TLC, CH_2_Cl_2_:MeOH:H_2_O = 7:3:0.1). The solution was cooled down to an ambient temperature, and acetic anhydride (20.0 mL) was added. The mixture was heated to 55 °C for 2 h. The solution was cooled down to an ambient temperature and concentrated under reduced pressure. The residue was dissolved in EtOAc (150 mL), and the organic solution was successively washed with an aqueous solution of HCl (2M, 50 mL), 10% NaHCO_3_ (50 mL) and 10% brine (50 mL), dried over anhydrous Na_2_SO_4_ and evaporated to afford crude per-*O*-acetylated dibromide (**4**). The residue was dissolved in anhydrous DMF (20.0 mL), and NaN_3_ (2.0 g, 30.8 mmol) was added, and the mixture was heated to 70 °C overnight. The mixture was cooled down to room temperature and diluted with EtOAc (150 mL). The organic solution was extracted with 10% brine (2 × 100 mL), dried over anhydrous Na_2_SO_4_ and evaporated. The crude material was purified by column chromatography on silica gel using 1% acetone in dichloromethane as the eluent to afford compound **5** in its pure form and as a white foam (2.55 g, 67%). ^1^H NMR (400 MHz, CDCl_3_) *δ* 5.60 (d, *J* = 3.7 Hz, 1H, H-1), 5.44–5.33 (m, 2H, H-3 + H-3’), 5.28–5.21 (dd, *J* = 5.9, 5.9 Hz, 1H, H-4’), 4.96 (dd, *J* = 10.2, 9.4 Hz, 1H, H-4), 4.79 (dd, *J* = 10.4, 3.6 Hz, 1H, H-2), 4.21–4.15 (m, 2H, H-1a’ + H-5), 4.13 (d, *J* = 12.3 Hz, 1H, H-1b’), 4.08 (ddd, *J* = 7.6, 5.8, 4.2 Hz, 1H, H-5’), 3.62 (dd, *J* = 13.1, 7.5 Hz, 1H, H-6a’), 3.48 (dd, *J* = 13.1, 4.2 Hz, 1H, H-6b’), 3.36 (dd, *J* = 13.4, 2.8 Hz, 1H, H-6a), 3.27 (dd, *J* = 13.4, 5.9 Hz, 1H, H-6b’), 2.10 (s, 3H, OAc), 2.03 (s, 3H, OAc), 2.025 (s, 3H, OAc), 2.02 (s, 3H, OAc), 1.97 (s, 3H, OAc) and 1.94 (s, 3H, OAc). ^13^C NMR (101 MHz, CDCl_3_) *δ* 169.94 (×2, C=O), 169.88 (C=O), 169.87 (C=O), 169.50 (C=O), 169.43 (C=O), 103.95 (C-2), 90.22 (C-1), 80.09 (C-5), 75.89 (C-3’), 75.66 (C-4’), 70.18 (C-2), 69.56 (C-5), 69.21, 69.18 (C-3, C-4), 62.51 (C-1’), 52.72 (C-6’), 50.99 (C-6), 20.56 (Ac), 20.48 (Ac), 20.47 (Ac), 20.44 (Ac), 20.41 (Ac) and 20.33 (Ac). HRMS (ESI-QTOF, positive) *m*/*z* calculated for C_24_H_32_N_6_O_15_Na (M + Na^+^): 667.1818; found 667.1829.

**2,3,4,1**′**,2**′**,3**′**-Hexa-*O*-acetyl-6,6**′**-dideoxy-6,6**′**-(4-((bis(2-methoxycarbonylethyl)amino)methyl)-1*H*-1,2,3-triazol-1-yl)sucrose (7).** Compound **5** (1.0 g, 1.55 mmol) and *N,N*-bis(methoxycarbonylmethyl)propargylamine (**6**) (1.18 g, 5.92 mmol, 1.91 equivalents per N_3_) were dissolved in acetone (25.0 mL) under an argon atmosphere. *N,N*-di*iso*propylethylamine (90 μL, 0.51 mmol) and a catalytic amount of CuI (~60 mg, 0.31 mmol) were added. After stirring the solution at an ambient temperature for 2 days, the mixture was concentrated under reduced pressure. The crude mixture was then dissolved in EtOAc (100 mL) and washed with a saturated solution of EDTA (2 × 30 mL). The organic solution was dried over anhydrous Na_2_SO_4_ and concentrated under reduced pressure. The crude residue was then purified by column chromatography on silica gel using a gradient of 1→2% MeOH–CH_2_Cl_2_ as the eluent to afford the desired conjugate **7** (1.34 g, 1.28 mmol, 83% yield). ^1^H NMR (400 MHz, CDCl_3_) *δ* 7.67 (s, 1H, H-4_1,2,3-triazole), 7.57 (s, 1H, H-4_1,2,3-triazole), 5.53 (d, *J* = 3.6 Hz, 1H, H-1), 5.38 (dd, *J* = 10.4, 9.4 Hz, 1H, H-3), 5.26 (d, *J* = 4.7 Hz, 1H, H-3’), 5.16 (dd, *J* = 4.6, 4.6 Hz, 1H, H-4’), 4.76 (dd, *J* = 9.7, 9.7 Hz, 1H, H-4), 4.70 (dd, *J* = 10.4, 3.6 Hz, 1H, H-2), 4.53 (dd, *J* = 14.1, 5.6 Hz, 1H, H-6a’), 4.49–4.34 (m, 3H, H-5 + H-6a + H-6b), 4.30 (dd, *J* = 14.1, 7.3 Hz, 1H, H-6b’), 4.19 (ddd, *J* = 7.4, 5.6, 4.5 Hz, 1H, H-5’), 4.00–3.84 (m, 6H, 2 × 1,2,3-triazol-C*H*a*H*bN + 2 × 1,2,3-triazol-C*H*a*H*bN + H-1a’ + H-1b’), 3.58 (s, 6H, 2 × CO_2_Me), 3.578 (s, 6H, 2 × CO_2_Me), 3.48 (s, 4H, 2 × NC*H*_2_CO_2_Me), 3.44 (s, 4H, 2 × NC*H*_2_CO_2_Me), 2.07 (s, 3H, Ac), 2.03 (s, 3H, Ac), 1.98 (s, 3H, Ac), 1.95 (s, 3H, Ac), 1.94 (s, 3H, Ac) and 1.92 (s, 3H, Ac). ^13^C NMR (101 MHz, CDCl_3_) *δ* 171.25 (×2, C=O), 171.21 (×2, C=O), 169.97 (C=O), 169.79 (C=O), 169.76 (C=O), 169.63 (C=O), 169.59 (C=O), 169.19 (C=O), 145.03 (C-3_1,2,3-triazole), 144.81 (C-3_1,2,3-triazole), 124.77 (C-4_1,2,3-triazole), 124.23 (C-4_1,2,3-triazole), 104.81 (C-2’), 90.25 (C-1), 80.23 (C-5’), 75.95 (C-4’), 75.33 (C-3’), 70.11 (C-2), 69.41 (C-4), 69.06 (C-5), 68.85 (C-3), 61.23 (C-1’), 54.12 (×2, 2 × N*C*H_2_CO_2_Me), 54.09 (×2, 2 × N*C*H_2_CO_2_Me), 51.69 (C-6’), 51.46 (CO_2_*Me*), 51.44 (CO_2_*Me*), 50.35 (C-6), 48.85 (1,2,3-triazole-*C*HaHbN), 48.80 (1,2,3-triazole-*C*HaHbN), 20.58 (Ac), 20.46 (Ac), 20.43 (Ac), 20.33 (×2, 2 × Ac) and 20.28 (Ac). HRMS (ESI-QTOF, positive) *m*/*z* calculated for C_42_H_58_N_8_O_23_Na (M + Na^+^): 1065.3512; found 1065.3502.

**6,6′-Dideoxy-6,6′-(4-((bis(2-carboxyethyl)amino)methyl)-1*H*-1,2,3-triazol-1-yl)sucrose, disodium salt (2).** Compound **7** (480 mg, 0.460 mmol) was dissolved in anhydrous MeOH (10.0 mL), and a solution of NaOMe in MeOH (1.0 M, 0.10 mL) was added. The reaction was then stirred under argon overnight and concentrated under reduced pressure. The obtained residue was dissolved in H_2_O (5.0 mL). NaOH (110 mg, 2.75 mmol, 1.5 equivalents per carboxylic ester) was then added to the solution, and the mixture was heated at 80 °C under argon overnight. The mixture was then neutralized with dry ice and concentrated under reduced pressure, and the residue was purified by gel filtration chromatography on Sephadex LH-20 using Millipore H_2_O as the eluent to afford the desired compound (2), which was subsequently freeze-dried as a white solid (310 mg, 0.398 mmol, 86% yield). ^1^H NMR (400 MHz, D_2_O) *δ* 8.37 (s, 1H, H-4_1,2,3-triazole), 8.32 (s, 1H, H-4_1,2,3-triazole), 5.44 (d, *J* = 3.7 Hz, 1H, H-1), 4.93 (dd, *J* = 15.0, 2.7 Hz, 1H, H-6a), 4.85 (overlapped with HDO, 1H, H-6b), 4.73 (s, 2H, 1,2,3-triazole-C*H*a*H*bN), 4.71–4.55 (m, 3H, 1,2,3-triazole-C*H*a*H*bN + H-6a’), 4.36 (ddd, *J* = 9.6, 6.7, 2.6 Hz, 1H, H-5), 4.30–4.10 (m, 3H, H-3’ + H-6b’ + H-5’), 4.00–3.85 (m, 9H, H-4’ + 4 × NCH_2_CO_2_), 3.82 (dd, *J* = 9.5, 9.5 Hz, 1H, H-3), 3.69–3.52 (m, 3H, H-1a’ + H-2 + H-1b’) and 3.26 (dd, *J* = 9.6, 9.6 Hz, 1H, H-4). ^13^C NMR (101 MHz, D_2_O) *δ* 170.13 (×2, C=O), 136.77 (C-3_1,2,3-triazole), 136.70 (C-3_1,2,3-triazole), 129.03 (C-4_1,2,3-triazole), 128.19 (C-4_1,2,3-triazole), 104.18 (C-2’), 92.30 (C-1), 78.65 (C-5’), 75.95 (C-3’), 75.36 (C-4’), 72.06 (C-3), 70.98 (C-5), 70.76 (C-4), 70.64 (C-2), 61.10 (C-1’), 56.31 (×2, 2 × N*C*H_2_CO_2_), 56.29 (×2, 2 × N*C*H_2_CO_2_), 52.85 (C-6’), 51.47 (C-6), 48.95 (1,2,3-triazole-*C*H_2_N) and 48.81 (1,2,3-triazole-*C*H_2_N). HRMS (ESI-QTOF, negative) *m*/*z* calculated for C_26_H_35_N_8_O_17_ (M-H)^−^: 733.2282; found 733.2289; *m*/*z* calculated for C_26_H_35_N_8_O_17_ (M-2H)^2−^: 366.1105; found 366.1114.

### 4.2. Preparation of Ligand **2**/Gd(III) Complex

Ligand **2** (14.3 mg, 19 μmol) was dissolved in Milli-Q water (1 mL) to obtain a solution (19 mM concentration). The pH was adjusted between 6 and 7 with a 0.01 M NH_4_OH solution. A volume (1 μL) of the GdCl_3_ solution (21 mM, 1.1 equivalent) was added to the solution of ligand **2**. The pH was adjusted several times to be maintained between 6 and 7 during the reaction. The reaction mixture was stirred for 24 h. Thereafter, the solution was dialyzed for 24 h in a spectrum Por (MWCO: 0.5 kDa) dialysis tube closed at both ends by Mohr clamps and suspended in a crystallizer filled with Milli-Q water (changed regularly every 2 h). The solution was then lyophilized to afford a white solid (17.1 mg), which is hygroscopic. 

### 4.3. Ligand **2** Protonation Studies

All solutions were prepared with Milli-Q water and degassed by argon saturation in order to remove dissolved CO_2_. A total of 0.01 M HCl was prepared from standard Titrisol (Merck, Rahway, NJ, USA) and adjusted to I = 0.1 M with NMe_4_Cl. This solution was used to calibrate the Metrohm combined glass microelectrode. Carbonate-free NMe_4_OH solution (ca. 0.05 M) was standardized against potassium hydrogen phthalate (RPE, Carlo Erba) by potentiometry, recording e.m.f. (mV) after each base addition.

Alkali metal ions have strong tendencies to form partially dissociated complexes in solutions with carboxylate ions. The affinity of this association tends to increase with the number of carboxylic acid groups of the acid anion. Therefore, potentiometric studies were performed using tetramethylammonium chloride (NMe_4_Cl) as a background electrolyte. 

The ionic product of water was determined by titrating an acetic solution in 0.1 M NMe_4_Cl at different concentrations. Under these conditions, pKw = 13.78. This value was used in the calculations.

Automatic titrations were carried out with a 905 Metrohm Titrando apparatus equipped with an 800 Dosino 2 mL burette connected to a computer. The delivery of the titrant, data acquisition and monitoring for e.m.f. stability were controlled by Tiamo 2.2 software. The burette is capable of delivering titrant solution at an increment of 0.2 µL volumes. Sample solutions were titrated in a double-walled, glass-reaction vessel fitted with a sealed lid containing ports for the glass combination electrode (Metrohm #6.0234.100), Teflon anti-diffusion burette tip and Teflon gas line. All titrations were performed at 25 ± 0.2 °C in the thermostatic cell using a thermocirculator bath (Julabo HE cryostat) under an argon stream to avoid the dissolution of carbon dioxide. The electrode slope was checked by titration with an HCl solution. Linear behavior was observed in the range of pH 2 to 12.

All equilibrium measurements were carried out in 4.0 mL sample volumes under magnetic stirring. The ionic strength was adjusted to 0.1 N with NMe_4_Cl. The ligand stock solution was prepared by dissolution of a weighted amount of ligand in an appropriate amount of 0.1 M NMe_4_Cl solution. In our experiments, the ligand **2** concentration of the stock solution was equal to 10^−3^ M. Titrations with NMe_4_OH were carried out in the presence of extra HCl to ensure the initial protonation of carboxylate groups. The initial volume of the measured solution was 4.0 mL, and the ligand concentration varied from 1.25 × 10^−4^ to 1.0 × 10^−3^ M. Before the first point acquisition, the reaction cell was sealed, and the solution was stirred and sparged with argon for 10 min. Finally, the sample solution was titrated to ca. pH 12 with standardized NMe_4_OH in a monotonic mode. Increments of the base added were fixed at 4 µL. The electrode was monitored for up to 6 min or until its drift was less than 0.4 mV/min before recording an mV reading. The protonation constants of ligand **2** were determined from ten titrations.

The concentration of the ligand was calculated from potentiometric data (e.m.f.) during the determination of the protonation constants processed with the general computation program HYPERQUAD [30]. In HYPERQUAD, the stability constant, β (not log β), is the parameter that is refined, and by default, a standard deviation was labeled as excessive if it was more than 33% of the parameter value. Although this value is user-definable, the default value of 33% was used for this work.

### 4.4. Stability Constant Measurement

Stock solutions of metal chloride were prepared from commercially (gadolinium (III) chloride hydrate, Strem Chemicals) available reagents of purity higher than 99.9% in distilled water and diluted to the required concentrations. Their ionic strength was adjusted to 0.1 M with NMe_4_Cl. The exact concentration of metal chloride stock solution was determined by complexometric titrations with ethylenediaminetetraacetic acid disodium salt dihydrate (Titriplex^®^ III, Merck) in acetate buffer (50 mM, pH 5.8) using an xylenol orange indicator (Merck, ACS reagent) [31].

Stability constants of gadolinium–ligand **2** were determined from seventeen titrations using solutions containing 0 to over 1 equivalent of metal, with an initial ligand and HCl concentration equal, respectively, to 5 × 10^−4^ M and 3.45 × 10^−3^ M.

The same method was used for the metal–ligand stability constant determination. 

The computer program HYSS was used to obtain the speciation distribution curves [30,32].

### 4.5. Relaxivity Measurements

Relaxivity measurements were performed on a Brucker mq20 Minispec at 37 °C and 0.47 T (20 MHz). The relaxation time T_1_ of each concentration was determined using the “Inversion Recovery Pulse Sequence”. The resulting curves were adjusted to a monoexponential function to obtain T_1_. Relaxation values were measured three times, and the average was calculated. The concentration of gadolinium was verified by ICP-OES (iCAP Series 6000, Thermo Scientific, Waltham, MA, USA) at 336.2 nm. Calibration was performed in 2% HNO_3_ solutions with Gd concentrations ranging from 0.1 to 2.0 mg/L. The Gd complex was diluted in 2% HNO_3_ before analysis by ICP-OES.

## Figures and Tables

**Table 1:** Logarithmic values of the protonation constants of compound **2** determined by potentiometric titrations.

Species	log*βLH_h_*	*pKa*
LH	8.28	8.28
LH_2_	15.74	7.46
LH_3_	18.34	2.60

I = 0.1 M (NMe_4_Cl); T = 298 K; *pKw* = 13.78.

**Table 2:** Logarithmic values of the protonation constants of complex formed between ligand **2** and Gd(III) determined by potentiometric titrations.

$\mathrm{Species}\;(M_{m}L_{l}H_{h})$	*mlh*	logβ_Species_	*pK_a_*
GdLH_−1_	11−1	4.34	-
GdL	110	13.42	9.08
GdLH	111	16.44	3.02

I = 0.1 M (NMe_4_Cl); T = 298 K.

**Table 3:** Measured 1/T_1_ on different concentrations of ligand **2**/Gd(III) complex.

Concentration (mmol)	T_1_ (s)	1/T_1_ (s^−1^)
1.9	0.068	14.70588235
0.95	0.135	7.407407407
0.475	0.284	3.521126761
0.2375	0.49	2.040816327
0.11875	1.01	0.99009901

## Data Availability

The data are contained within the article.

## Supplementary Materials

The following supporting information can be downloaded at: https://www.mdpi.com/article/10.3390/molecules29194688/s1. Figure S1: ^1^H NMR spectrum of compound **5** in CDCl_3_, 400 MHz, 298 K; Figure S2: ^13^C NMR spectrum of compound **5** in CDCl_3_, 100 MHz, 298 K; Figure S3: ^1^H-^1^H 2D GCOSY NMR spectrum of compound **5** in CDCl_3_, 400 MHz, 298 K; Figure S4: ^1^H-^13^C 2D GHSQC NMR spectrum of compound **5** in CDCl_3_, 400 MHz, 298 K; Figure S5: ^1^H NMR spectrum of compound **7** in CDCl_3_, 400 MHz, 298 K; Figure S6: ^13^C NMR spectrum of compound **7** in CDCl_3_, 100 MHz, 298 K; Figure S7: ^1^H-^1^H 2D GCOSY NMR spectrum of compound **7** in CDCl_3_, 400 MHz, 298 K; Figure S8: ^1^H-^13^C 2D GHSQC NMR spectrum of compound **7** in CDCl_3_, 400 MHz, 298 K; Figure S9: ^1^H NMR spectrum of compound **2** in D_2_O, 400 MHz, 298 K; Figure S10: ^13^C NMR spectrum of compound **2** in D_2_O, 100 MHz, 298 K; Figure S11: ^1^H-^1^H 2D GCOSY NMR spectrum of compound **2** in D_2_O, 400 MHz, 298 K; Figure S12: ^1^H-^13^C 2D GHSQC NMR spectrum of compound **2** in D_2_O, 400 MHz, 298 K.
