# Supplementary material for: Synthesis of an Ethylenediaminetetraacetic Acid-like Ligand Based on Sucrose Scaffold and Complexation and Proton Relaxivity Studies of Its Gadolinium(III) Complex in Solution
Source: Molecules. 2024 Oct 3;29(19):4688. doi: 10.3390/molecules29194688 (PMC11478042; doi:10.3390/molecules29194688)

Supporting Information

Synthesis of an Ethylenediaminetetraacetic Acid-like Ligand Based on Sucrose Scaffold and Complexation and Proton Relaxivity Studies of Its Gadolinium (III) Complex in Solution

Ping Zhang,[1] Cécile Barbot,[2] Ramakrishna Gandikota,[2] Cenxiao Li,[1] Laura Gouriou,[2] Géraldine Gouhier,[2,*] Chang-Chun Ling[1,*]

[1]Department of Chemistry, University of Calgary, 2500 University Drive NW, Calgary, Alberta T2N 1N4, Canada

[2]Univ Rouen Normandie, INSA Rouen Normandie, CNRS, Normandie Univ, COBRA UMR 6014, INC3M FR 3038, F-76000 Rouen, France

E-mail: ccling@ucalgary.ca, Fax: +1-403-289-9488; geraldine.gouhier@univ-rouen.fr; Fax: +33-235522959

Contents

NMR Spectra 3

Figure S1: ^{1}H NMR spectrum of compound **5** in $CDCl_3$, 400 MHz, 298 K. 3

Figure S2: ^{13}C NMR spectrum of compound **5** in $CDCl_3$, 100 MHz, 298 K. 4

Figure S3: ^{1}H-^{1}H 2D GCOSY NMR spectrum of compound **5** in $CDCl_3$, 400 MHz, 298 K. 5

Figure S4: ^{1}H-^{13}C 2D GHSQC NMR spectrum of compound **5** in $CDCl_3$, 400 MHz, 298 K. 6

Figure S5: ^{1}H NMR spectrum of compound **7** in $CDCl_3$, 400 MHz, 298 K. 7

Figure S6: ^{13}C NMR spectrum of compound **7** in $CDCl_3$, 100 MHz, 298 K. 8

Figure S7: ^{1}H-^{1}H 2D GCOSY NMR spectrum of compound **7** in $CDCl_3$, 400 MHz, 298 K. 9

Figure S8: ^{1}H-^{13}C 2D GHSQC NMR spectrum of compound **7** in $CDCl_3$, 400 MHz, 298 K. 10

Figure S9: ^{1}H NMR spectrum of compound **2** in D_2O, 400 MHz, 298 K. 11

Figure S10: ^{13}C NMR spectrum of compound **2** in D_2O, 100 MHz, 298 K. 12

Figure S11: ^{1}H-^{1}H 2D GCOSY NMR spectrum of compound **2** in D_2O, 400 MHz, 298 K. 13

Figure S12: ^{1}H-^{13}C 2D GHSQC NMR spectrum of compound **2** in D_2O, 400 MHz, 298 K. 14

NMR Spectra

Figure S1: ^{1}H NMR spectrum of compound **5** in $CDCl_3$, 400 MHz, 298 K.

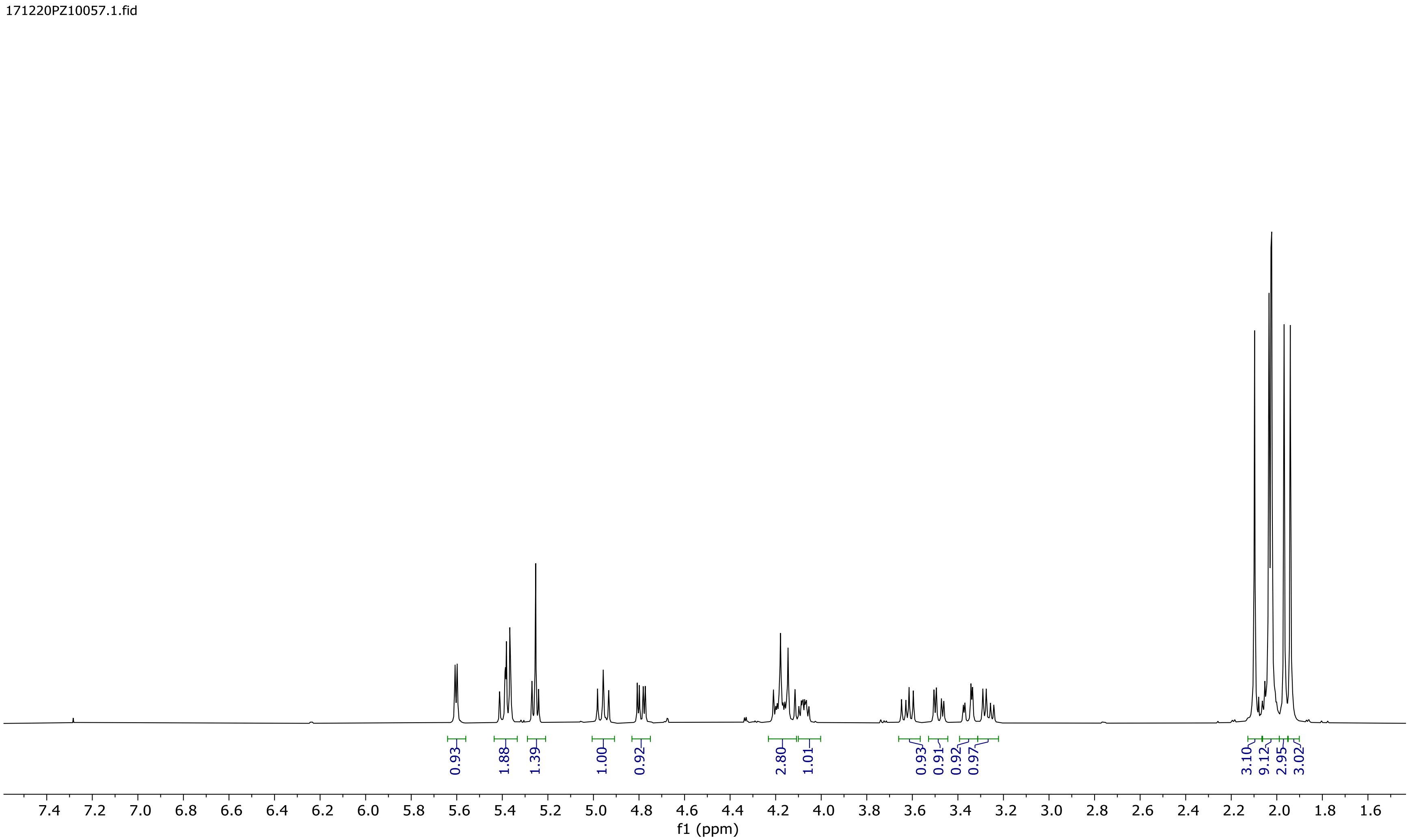

Figure S2: ^{13}C NMR spectrum of compound **5** in $CDCl_3$, 100 MHz, 298 K.

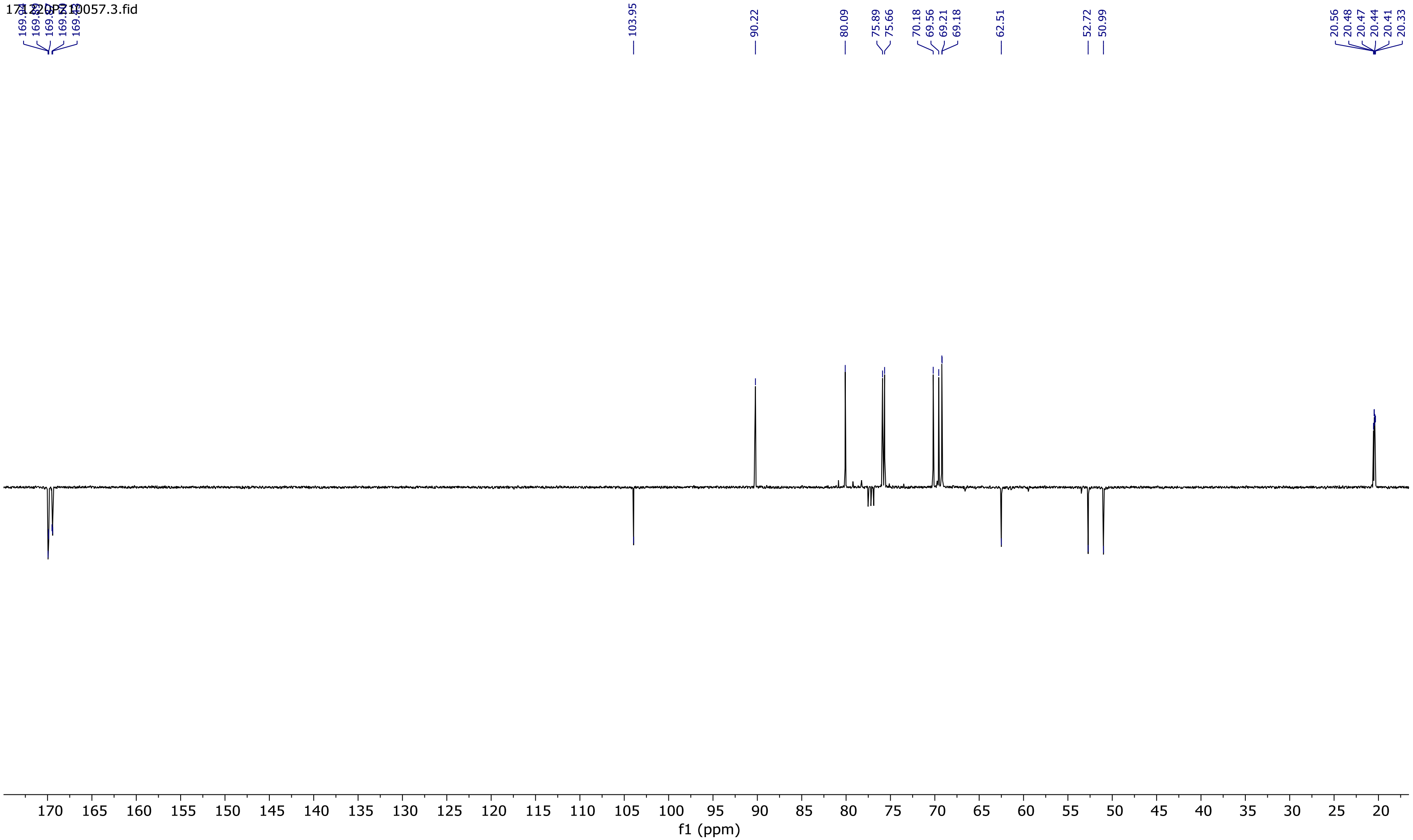

Figure S3: ^{1}H-^{1}H 2D GCOSY NMR spectrum of compound **5** in $CDCl_3$, 400 MHz, 298 K.

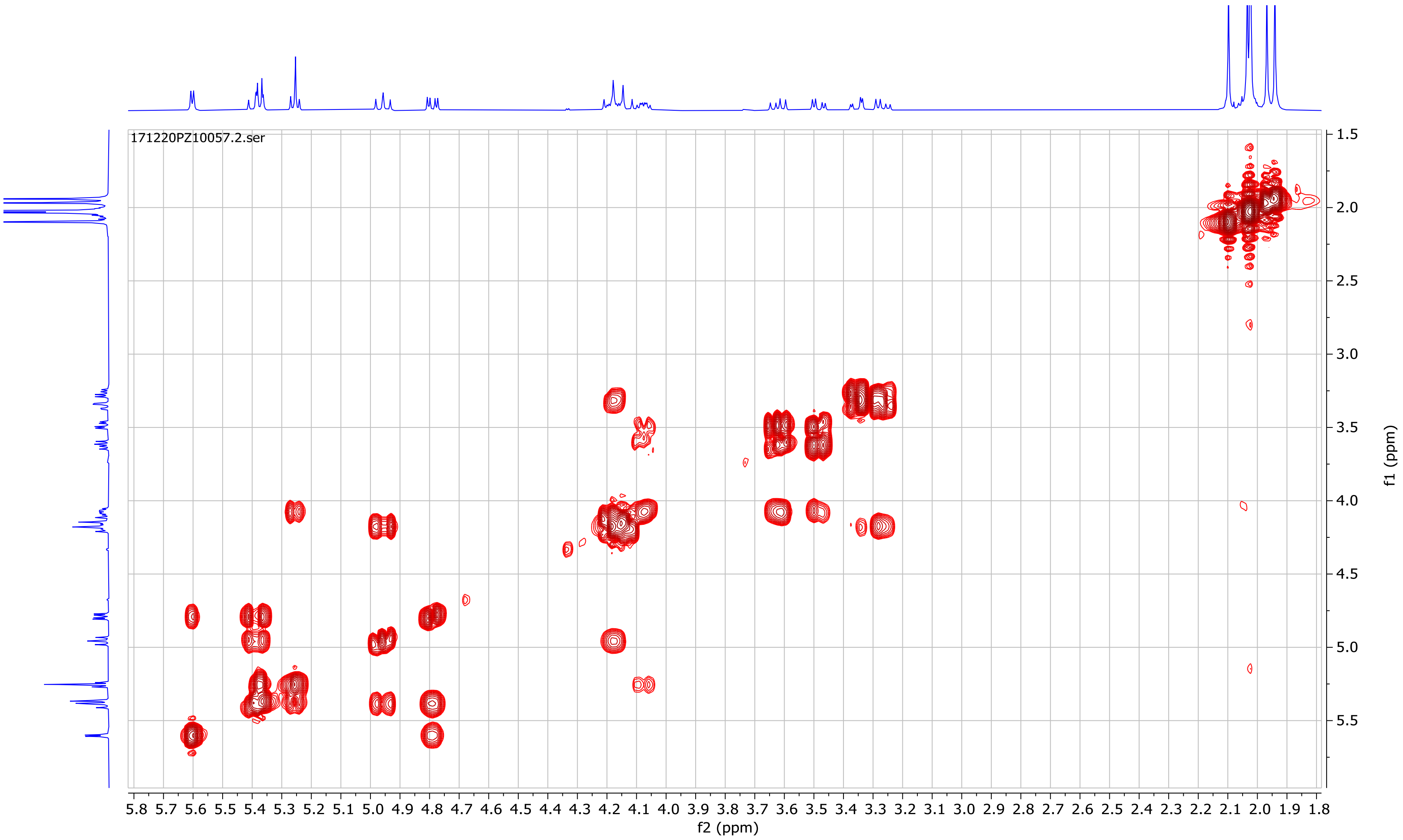

Figure S4: ^{1}H-^{13}C 2D GHSQC NMR spectrum of compound **5** in $CDCl_3$, 400 MHz, 298 K.

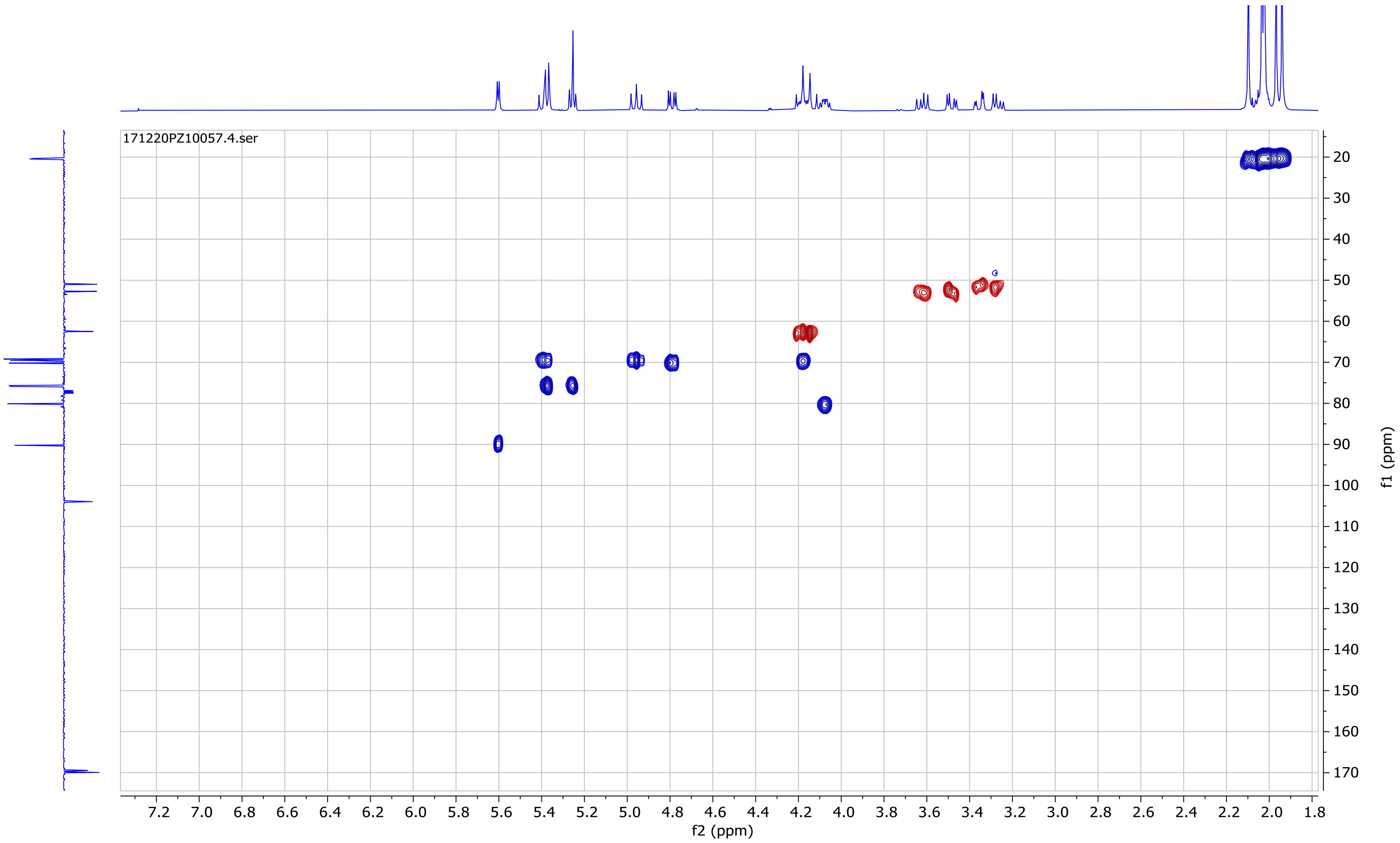

Figure S5: ^{1}H NMR spectrum of compound **7** in $CDCl_3$, 400 MHz, 298 K.

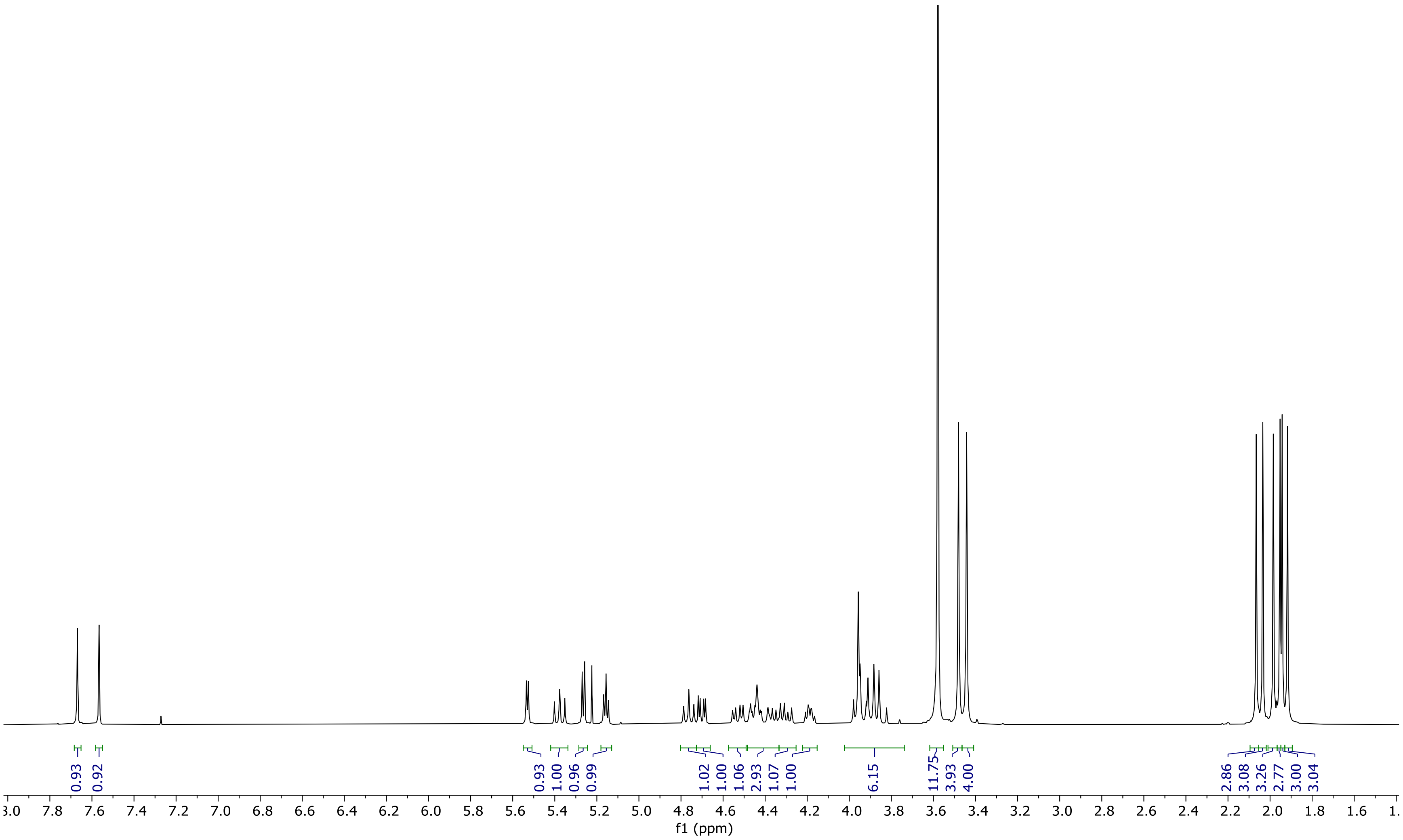

Figure S6: ^{13}C NMR spectrum of compound **7** in $CDCl_3$, 100 MHz, 298 K.

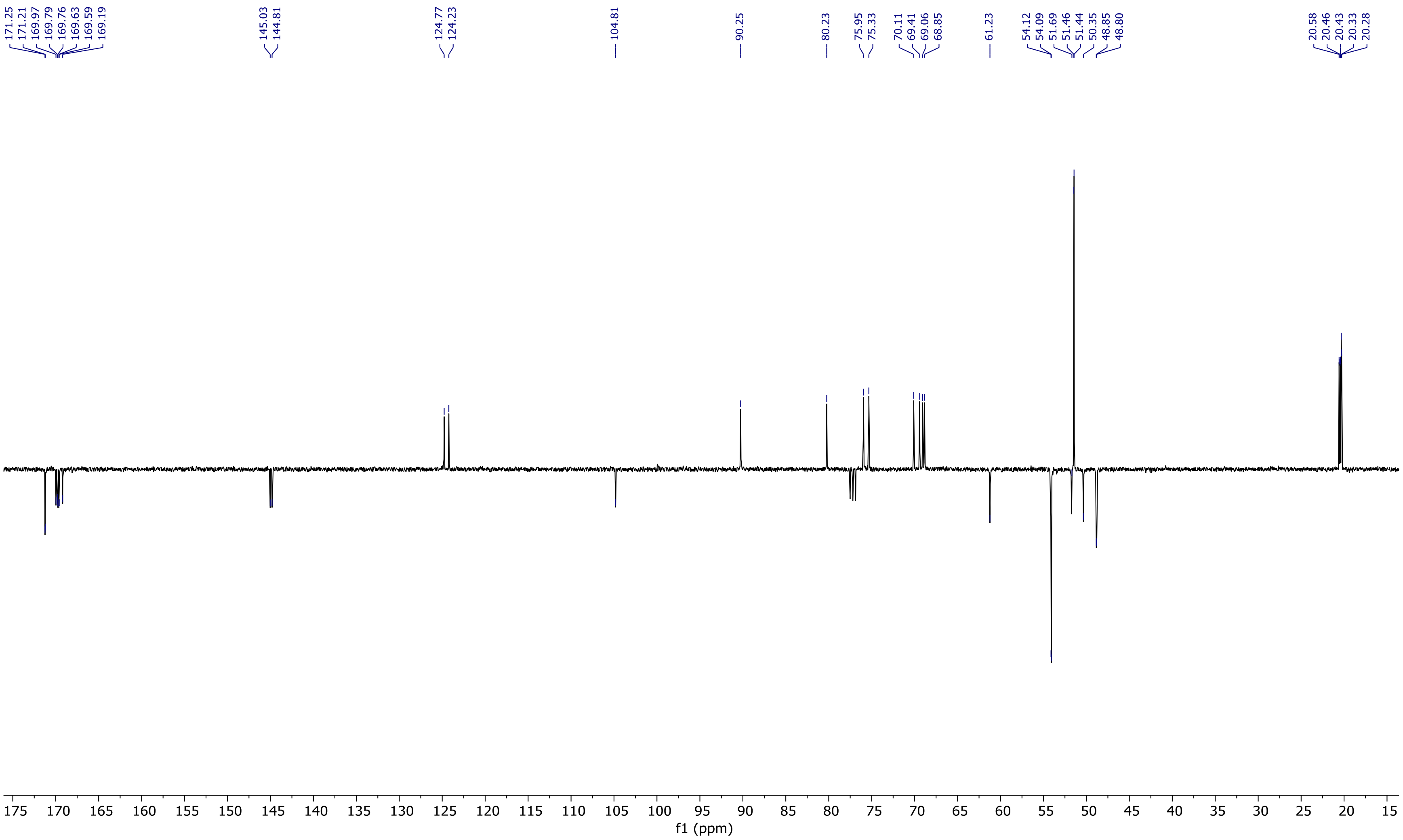

Figure S7: ^1H-^1H 2D GCOSY NMR spectrum of compound **7** in $CDCl_3$, 400 MHz, 298 K.

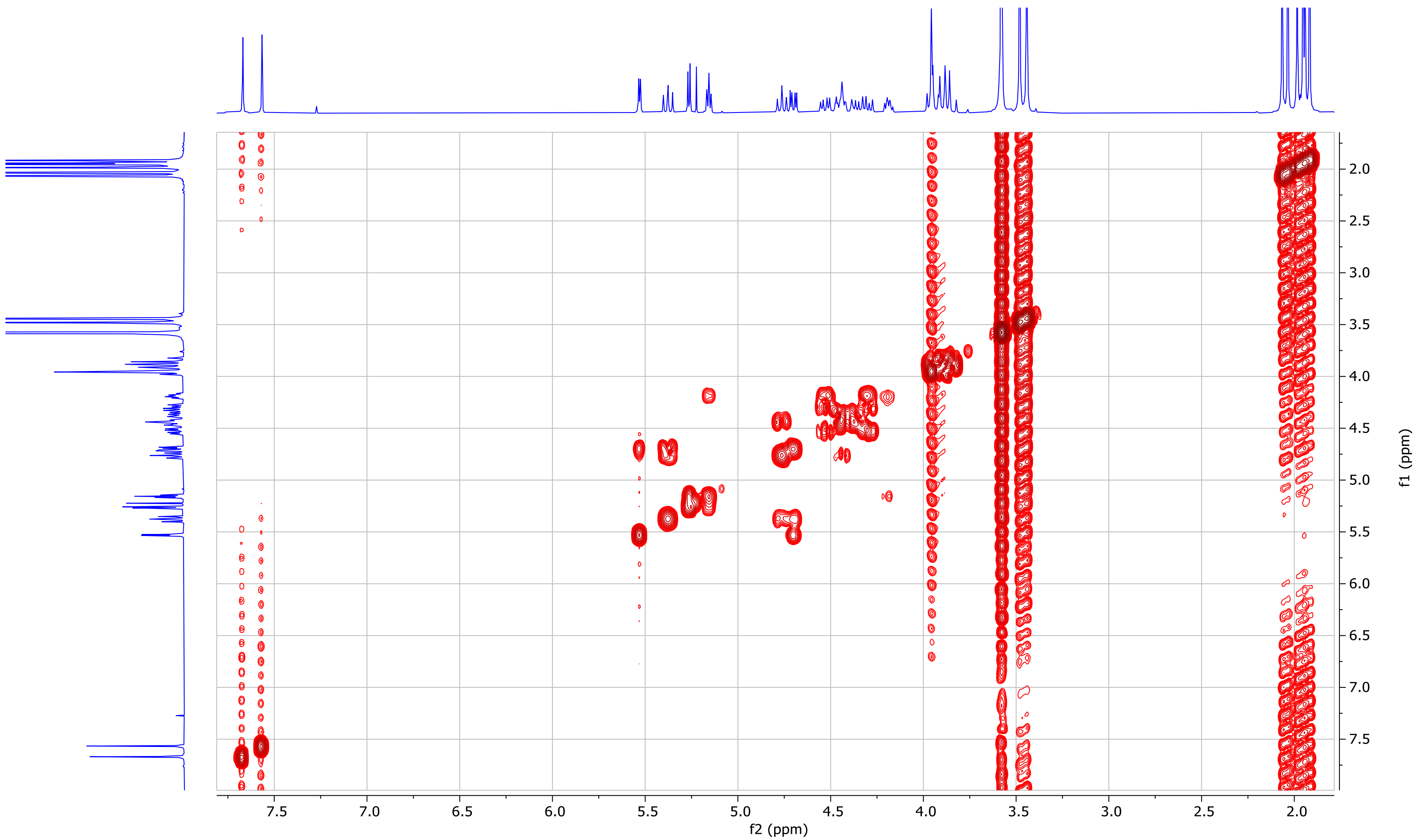

Figure S8: $^{1}H-^{13}C$ 2D GHSQC NMR spectrum of compound **7** in $CDCl_3$, 400 MHz, 298 K.

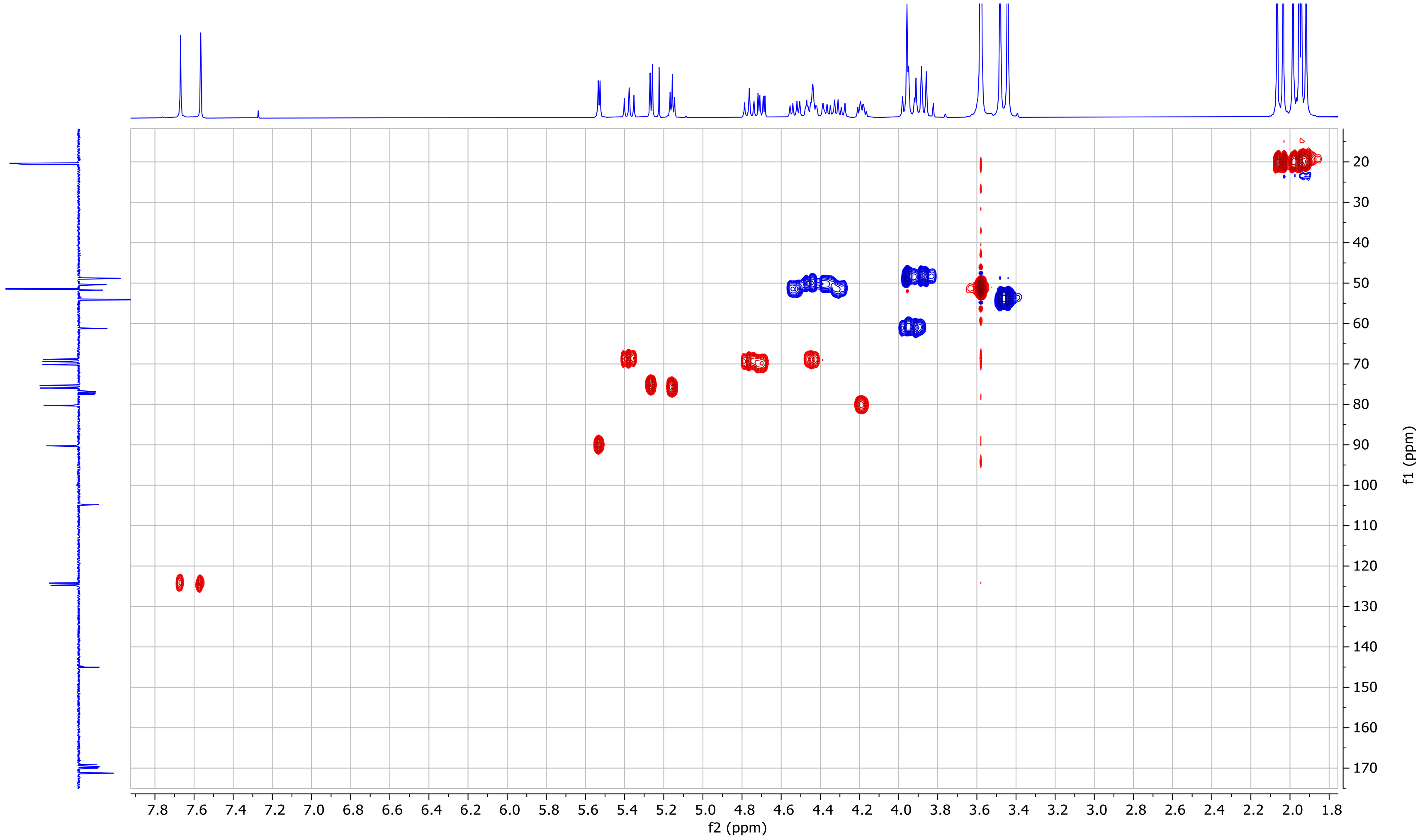

Figure S9: ^{1}H NMR spectrum of compound **2** in D_2O, 400 MHz, 298 K.

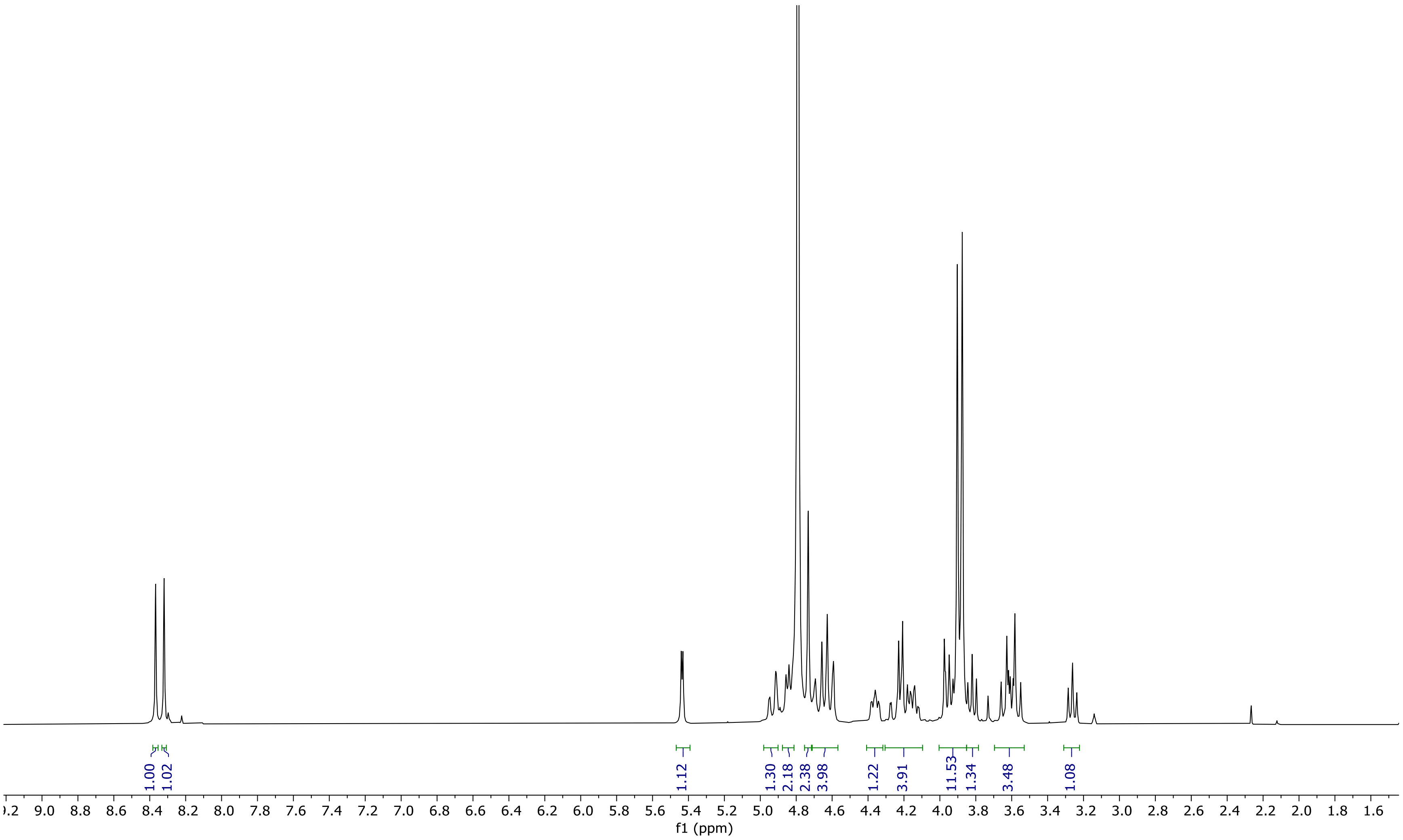

Figure S10: ^{13}C NMR spectrum of compound **2** in D_2O, 100 MHz, 298 K.

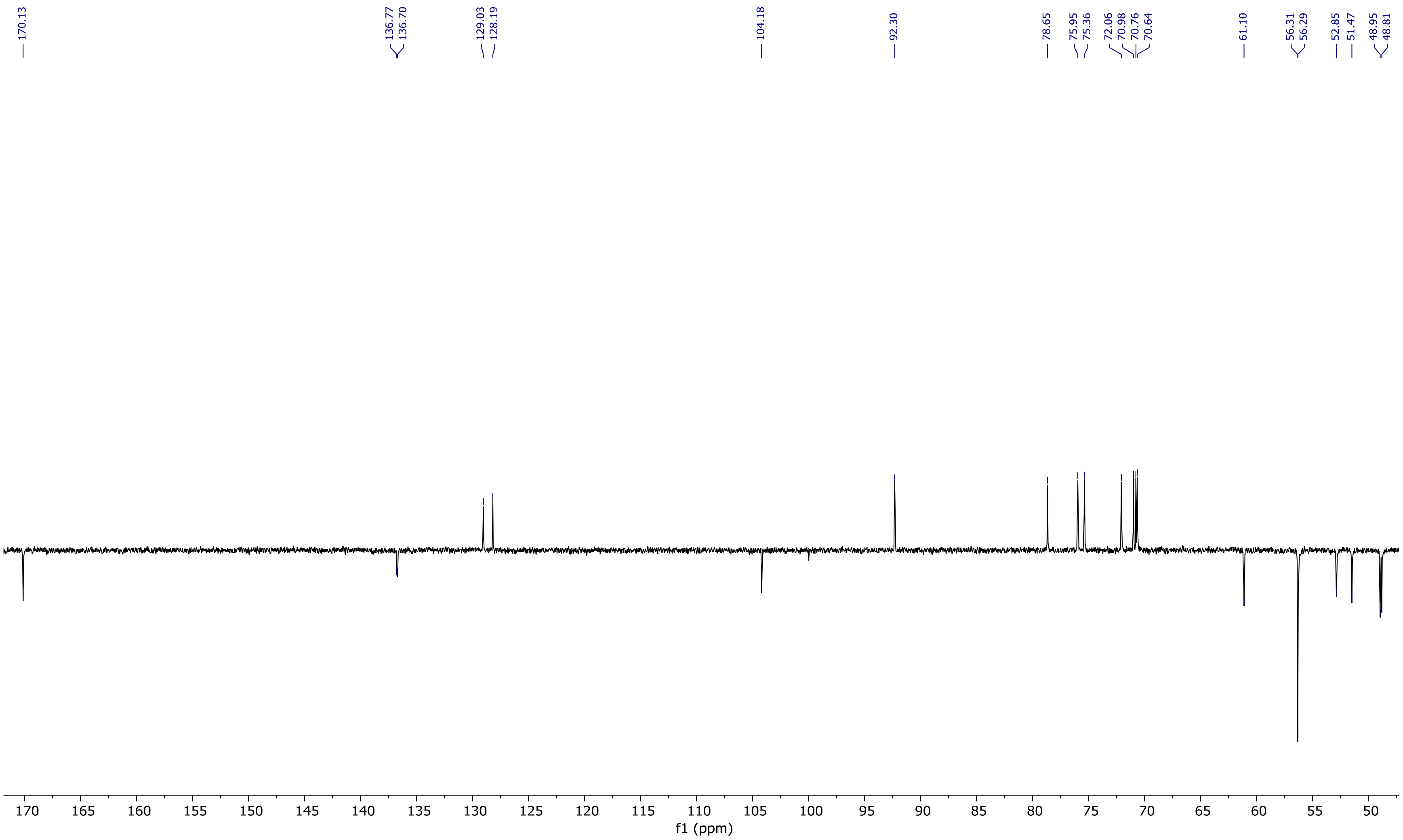

Figure S11: ^{1}H-^{1}H 2D GCOSY NMR spectrum of compound **2** in D_2O, 400 MHz, 298 K.

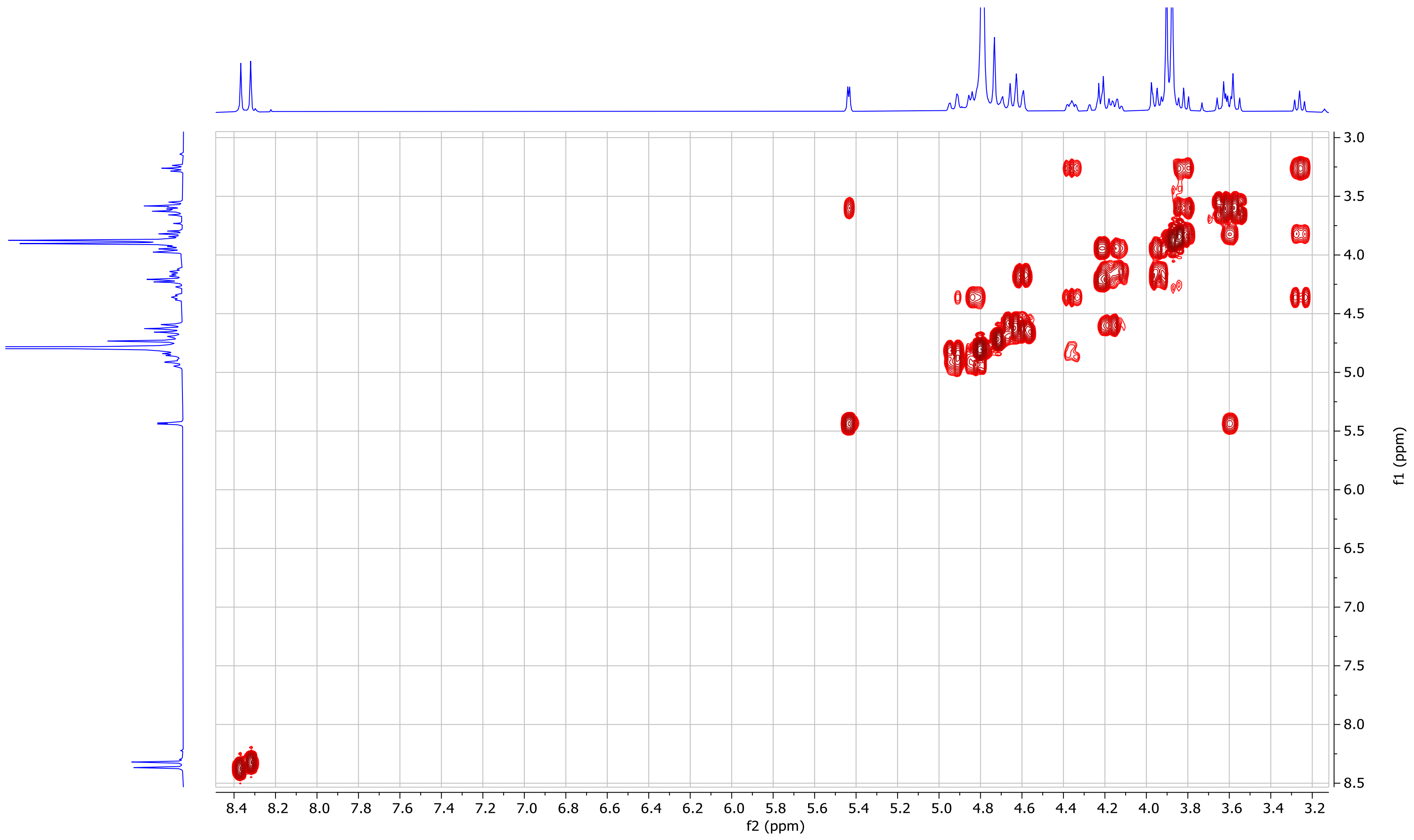

Figure S12: ^{1}H-^{13}C 2D GHSQC NMR spectrum of compound **2** in D_2O, 400 MHz, 298 K.

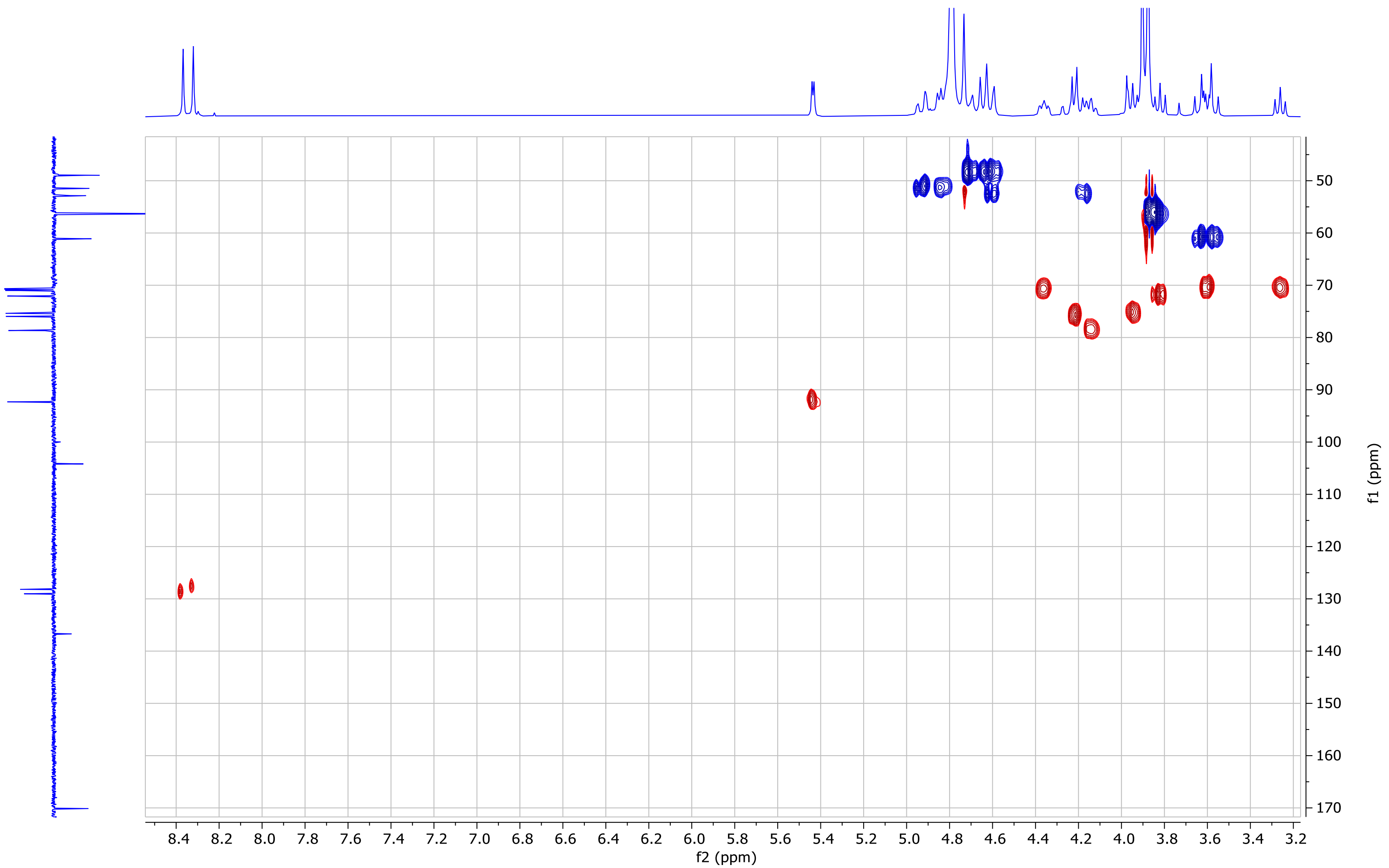

Supplement: Supplementary file 1 [file molecules-29-04688-s001.zip › molecules-3205666-supplementary.pdf]
